# Supplementary material for: Complement inhibition by a unique cluster of immunomodulatory outer surface proteins of Borrelia recurrentis
Source: Nat Commun. 2026 Apr 29;17:3900. doi: 10.1038/s41467-026-72359-y (PMC13128870; doi:10.1038/s41467-026-72359-y)
Supplement: Supplementary file 1 — Supplementary Information [file 41467_2026_72359_MOESM1_ESM.pdf]

Supplementary figure 1

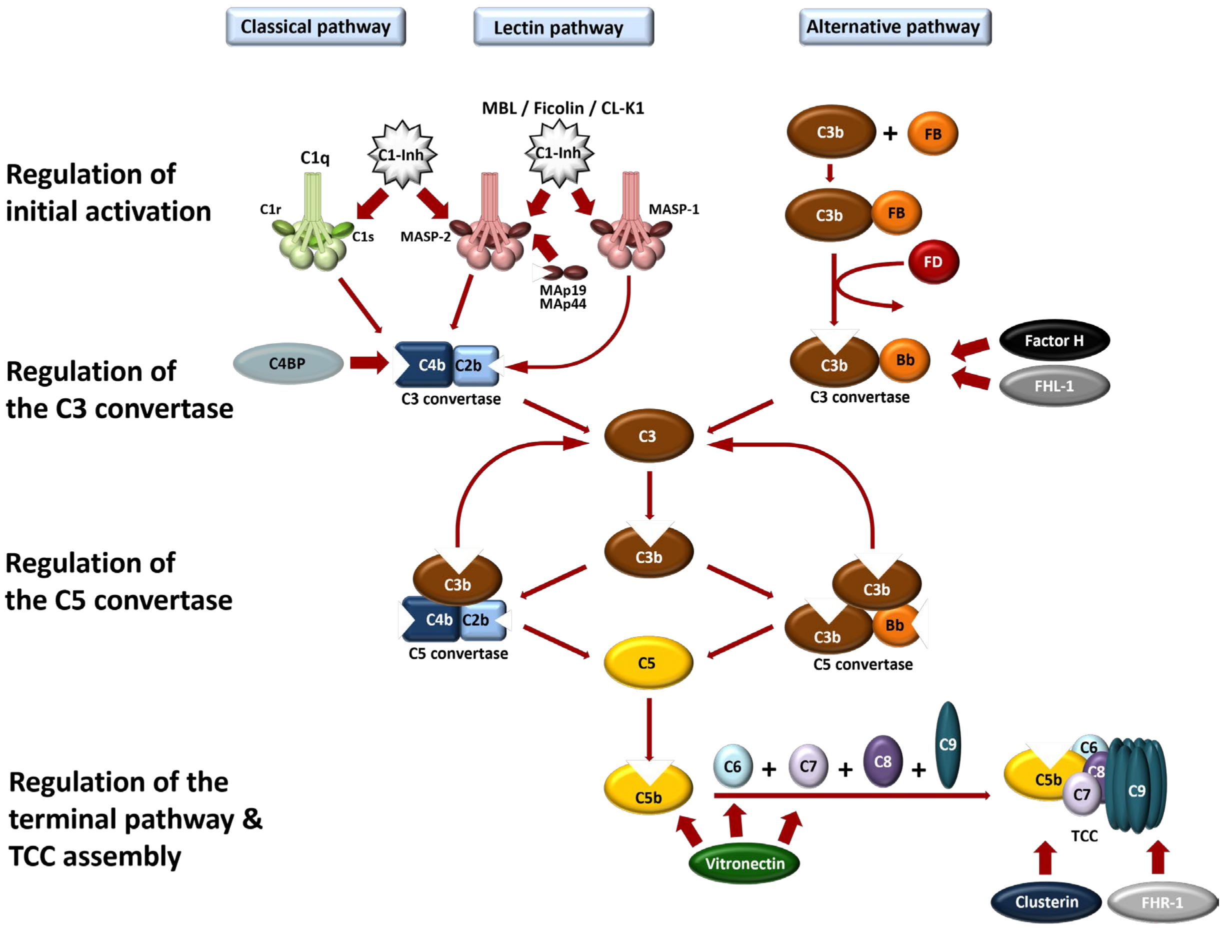

Activation of the complement cascade. Complement is activated by three pathways: the classical, lectin, and alternative pathway. The **classical pathway (CP)** is initiated by binding of the C1 complex consisting of one molecule of C1q, two molecules of C1r, and two molecules of C1s to immunoglobulins (IgM, IgG). Activated C1s cleaves C4 which, upon covalent binding to the target surface, cleaves C2 leading to the formation of the C3 convertase C4b2b. Activation of the **lectin pathway (LP)** is triggered by binding of a complex comprising MBL/Ficolins/Collectin CL-K1 associated with MASPs to a variety of carbohydrates of microbial origin. MASP-1 and MASP-2 are able to cleave C4 and then C2 to form the C3 convertase of the classical pathway. The **alternative pathway (AP)** is initiated by covalent binding of C3b molecules to foreign particles (opsonization). Surface-bound C3b molecules recruit Factor B (FB), a C2 homolog leading to the formation of a C3bB complex. Following cleavage of FB by Factor D (FD), the C3 convertase (C3bBb) of the AP is generated. This enzyme cleaves C3 into C3b and C3a Binding of C3b to the C3 convertases leads to the formation of the C5 convertase (C4b2b3b and C3bBb3b). Generation of C5b initiates the activation of the **terminal pathway (TP)** by sequential binding of C6, C7, C8, and C9 to C5b. Upon binding of multiple C9 molecules (C9 polymerization) the pore-forming membrane-attack complex (MAC) integrates into the membrane and leads to lysis of bacterial cells.

Complement is controlled by distinct soluble regulators at different activation levels of the cascade. C1-Inh inhibits complement initiation of the CP and LP. Factor H and FHL-1 serve as regulators of the AP by inactivating C3b in the presence of Factor I (FI). C4BP inhibits the formation of the C3 convertase of the CP and LP. Vitronectin, Clusterin, and FHR-1 prevent assembly of the MAC and integration of the complex into the bacterial membrane (Figure adopted from Kraiczy, P. (2015). Complement system in: Encyclopedia of Inflammatory Diseases. Doi 10.1007/978-3-0348-0620-6_219-1).

Supplementary figure 2

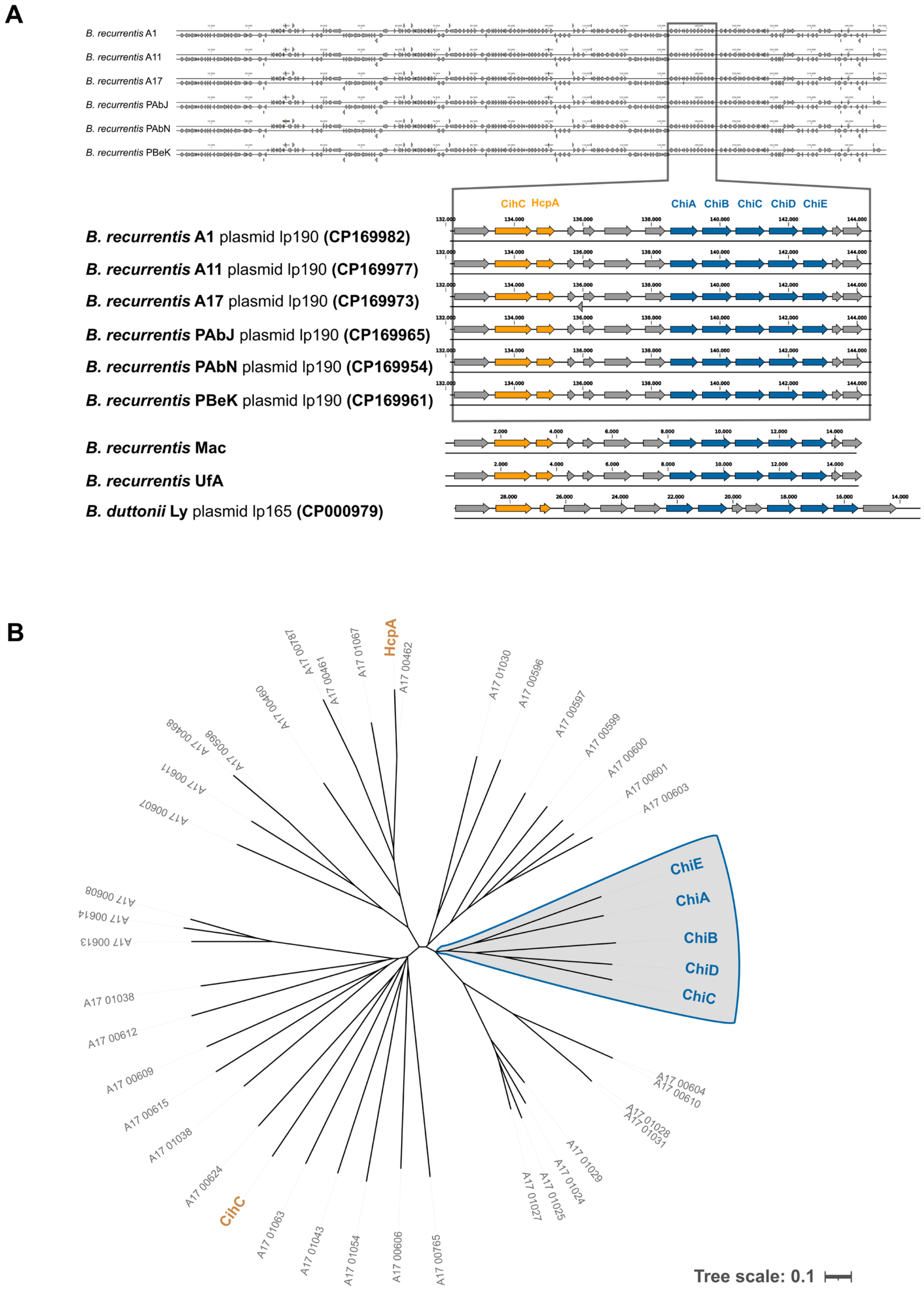

Identification of a unique cluster on lp190 of *B. recurrentis*. A unique cluster of homologous genes located on the large linear megaplasmid is widely conserved among of *B. recurrentis* strains. A genomic comparison of the large linear plasmid lp190 for all of the recently re-sequenced *B. recurrentis* strains highlights a conserved genomic synteny **(A)**. A close-up view on the locus harboring the gene cluster of interest (in blue) for different *B. recurrentis* strains and *B. duttonii* Ly as the most closely related Borrelia species is given below. No respective region was found in the genome of *B. hermsii* HS1 or *B. miyamotoi* CA17-2241 and LB2001. A genome wide search for proteins with local similarities was carried out for all these genes and deduced protein sequences were then aligned using Clustal Omega **(B)**.

Supplementary figure 3

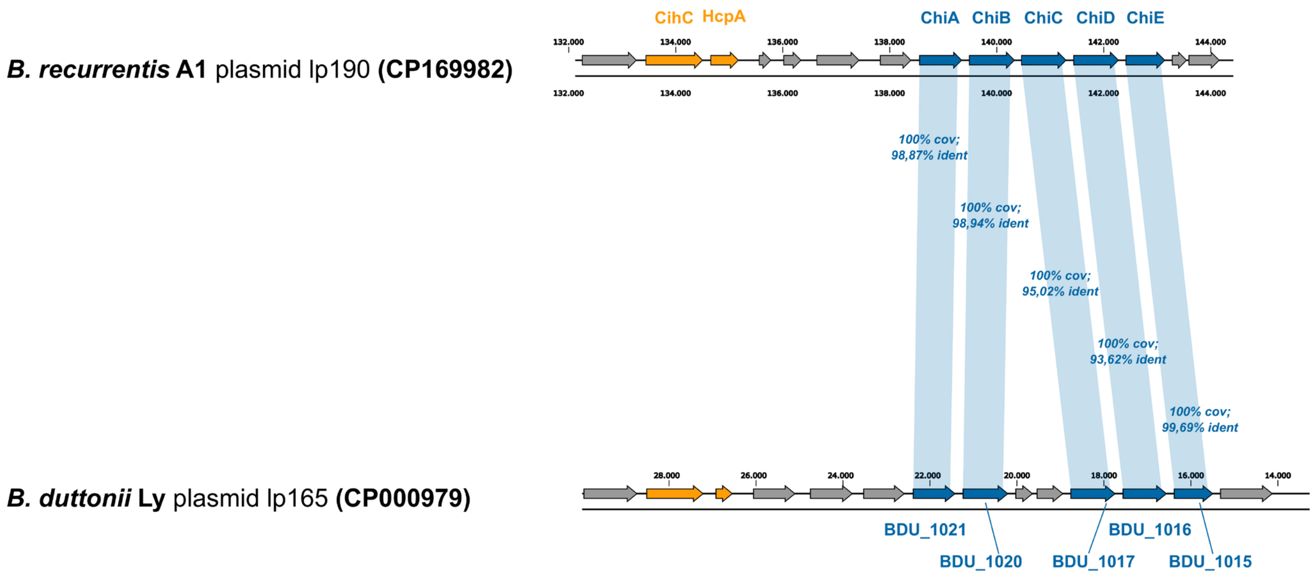

Comparative depiction of the Chi locus of *B. recurrentis* A17 and *B. duttonii* Ly. Homologous *chi* genes are colored in blue and the similarity of respective amino acid sequences is indicated as the result of pairwise BLASTp analyses which include percentage identity and percentage coverage.

Supplementary figure 4

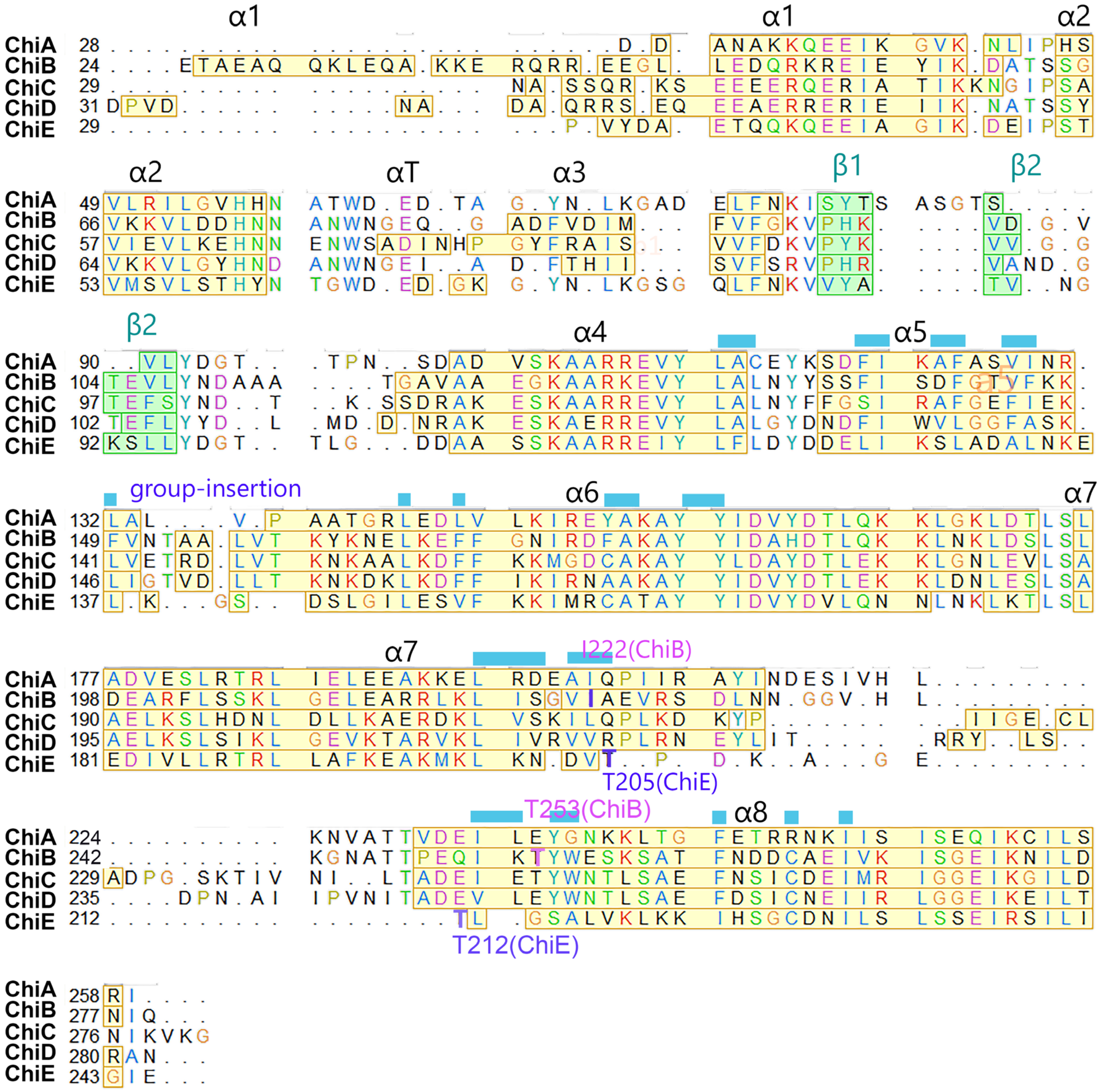

Sequence alignment and structural features of Chi proteins. Multiple sequence alignment of ChiA–ChiE generated using UCSF Chimera [74]. Predicted α-helices and β-sheets are highlighted in yellow and green, respectively. Residues forming the hydrophobic pocket are shown in cyan. The disordered N-terminal lipid anchor is omitted for clarity.

Supplementary figure 5

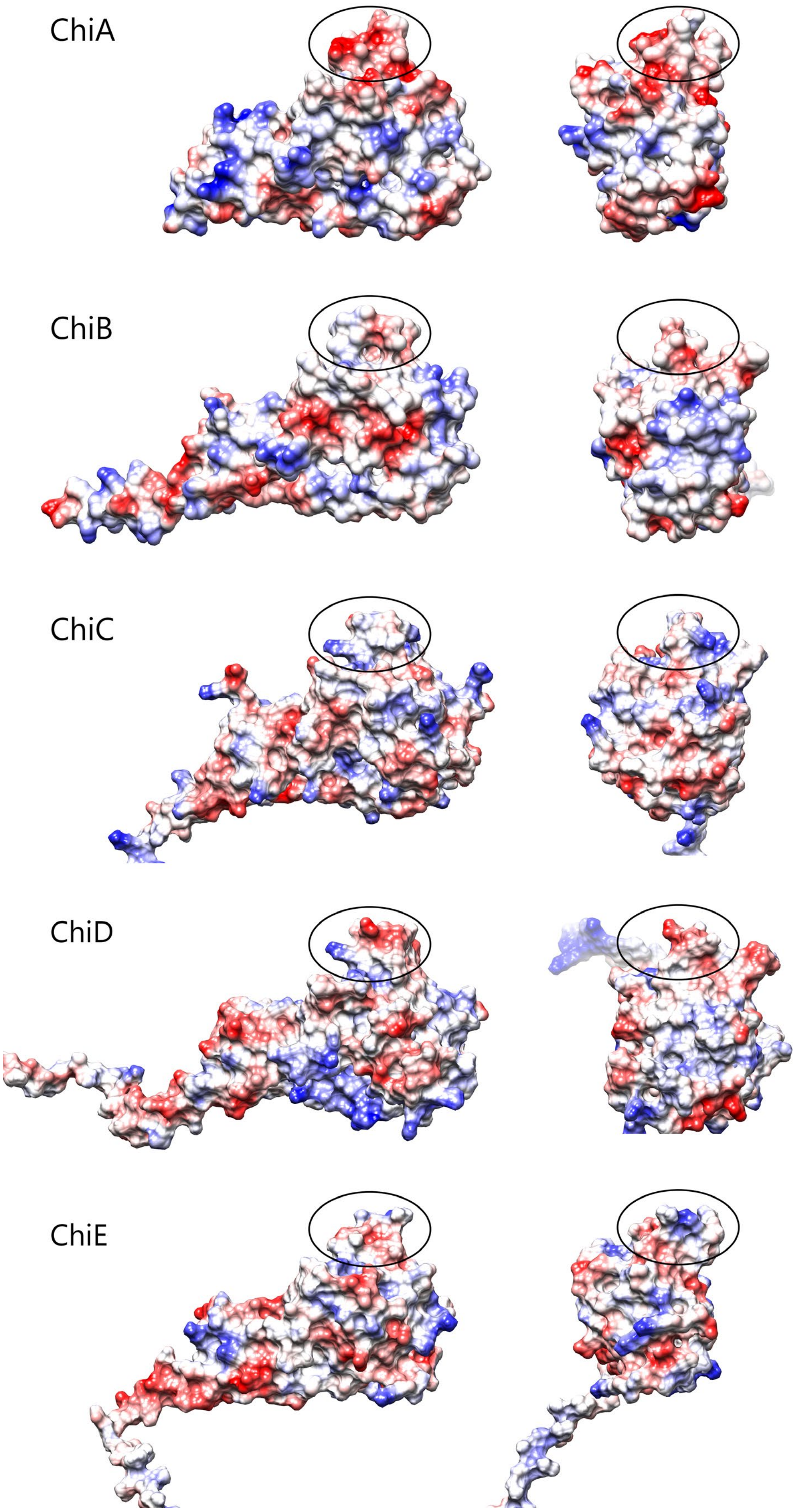

Surface representations of Chi proteins in two orientations. Electrostatic surface views of all five Chi proteins shown in two orientations rotated by 90°. Coulombic surface coloring ranges from red (−10) to blue (+10). Structures are aligned for consistent comparison.

Supplementary figure 6

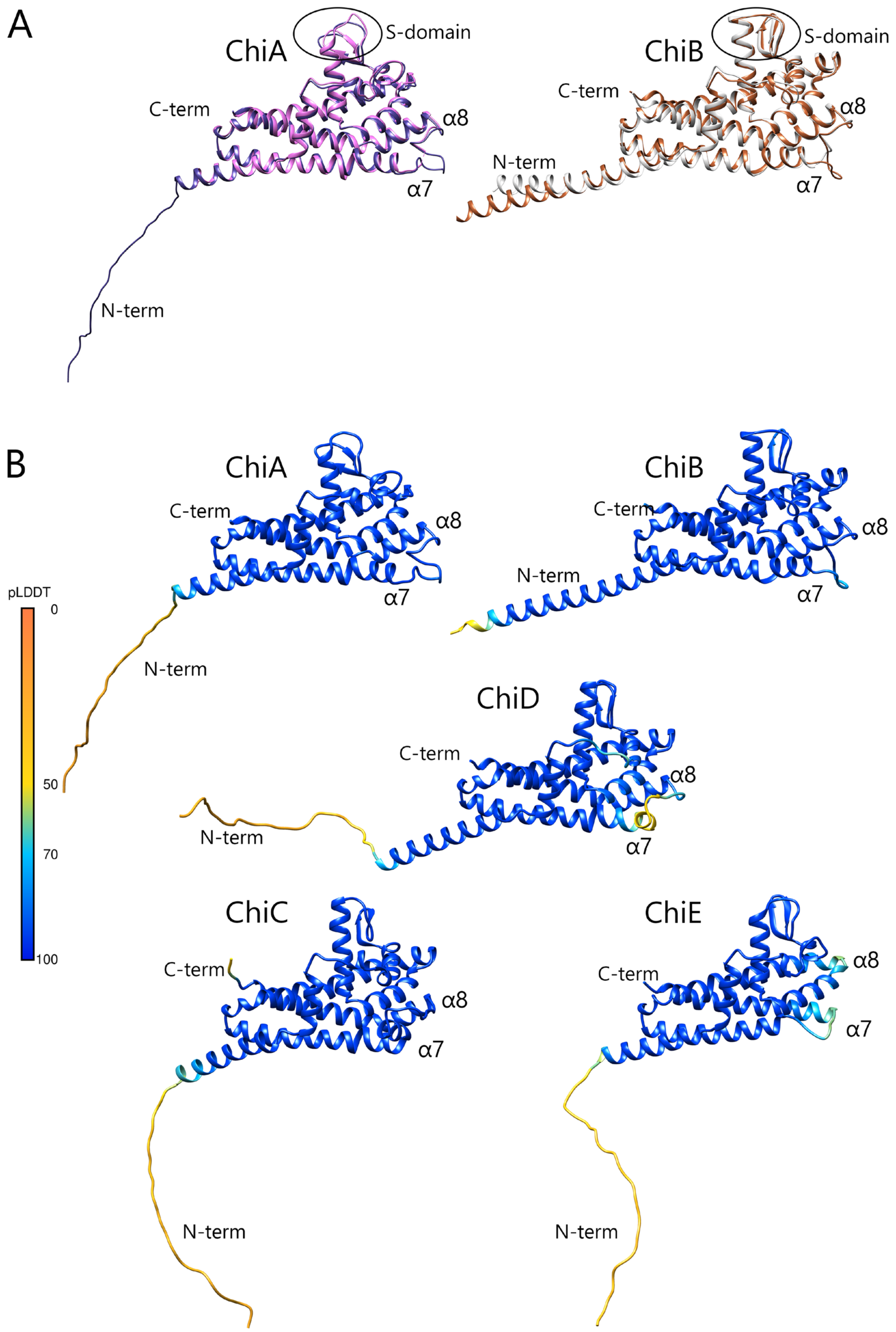

X-ray and AlphaFold2 (AF2) structures.(A) Structural alignment of X-ray structures (ChiA and ChiB) with AF2 models shows high similarity (ChiB: RMSD = 0.7 Å; ChiA: RMSD = 0.9 Å), with minor deviations at the α7–α8 junction and the N-terminal helix α1 in ChiB.(B) AF2 structures colored by predicted local distance difference test (pLDDT) using an AlphaFold-based color scheme: orange (<50, very low confidence), yellow (50–70, low confidence), light blue (70–90, confident), and deep blue (>90, very high confidence).

Supplementary figure 7

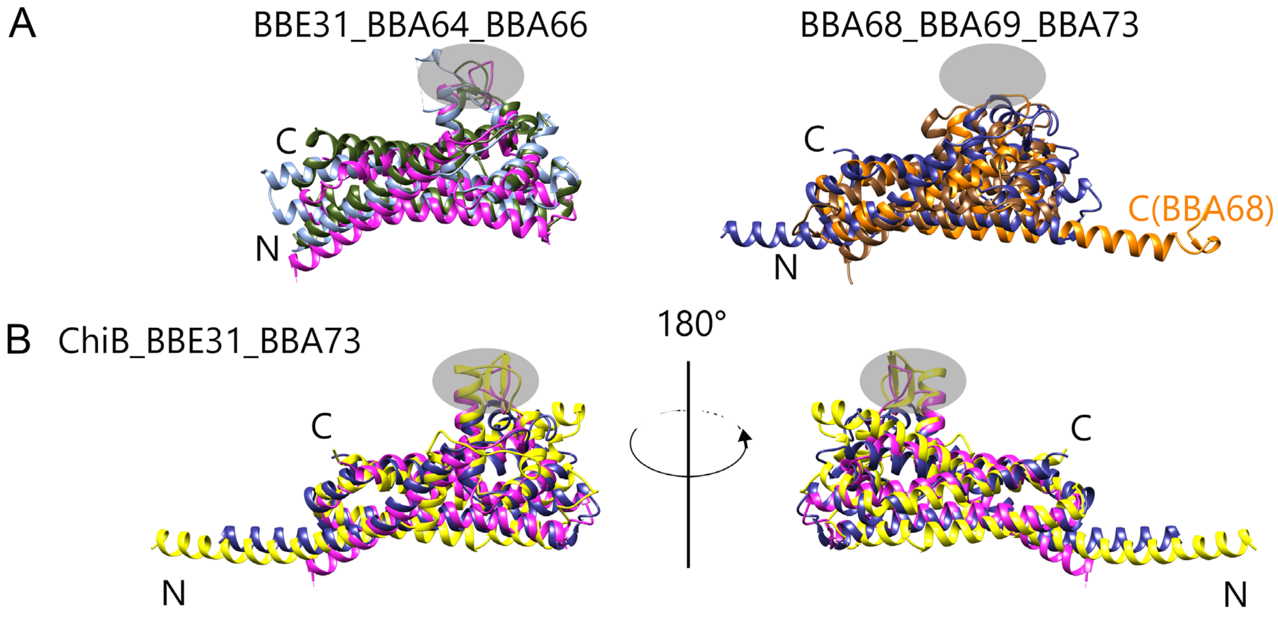

Structural comparison of ChiB with homologous PFam54_60 proteins. (A) Superposition of ChiB with PFam54_60 proteins using ChiA for orientation. The S-domain (grey ellipse), defined in ChiB, is indicated at equivalent positions. Left: BBE31 (magenta, 6fze), BBA64 (olive green, 4aly), and BBA66 (cornflower blue, 2yn7) contain a reduced S-domain with disordered loops instead of a β-hairpin. Right: BBA68 (orange, CspA, 4bl4), BBA69 (brown, 6qo1), and BBA73 (dark purple, 4axz) lack an S-domain due to shortened α2–α4 connections. (**B)** Structural alignment of ChiB (yellow), BBA31, and BBA73 (one per group) in two orientations shows notable shifts in helices α1, α2, α7, and α8 in ChiB. Helices in BBA31 and BBA73 align closely.

Supplementary figure 8

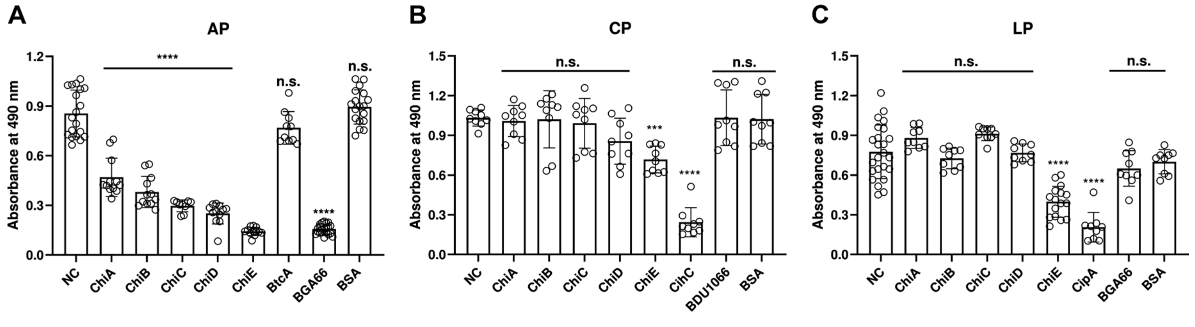

Assessment of the inhibitory capacity of Chi proteins on activation of the AP, CP, and LP. ELISA-based approaches were conducted to assess the inhibitory capacity of Chi proteins on activation of the AP **(A)**, CP **(B)**, and LP **(C)**. NHS pre-incubated with the purified Chi proteins or BSA were added to microtiter plates immobilized with LPS (AP), IgM (CP) or mannan (LP). Formation of the MAC was detected by using a monoclonal anti-C5b-9 antibody (n=9, technical replicates, mean, ±SD). ***, p ≤ 0.001; ****, p ≤ 0.0001, n.s., no statistical significance, one-way ANOVA with post-hoc Bonferroni multiple comparison test (confidence interval = 95%).

Supplementary figure 9

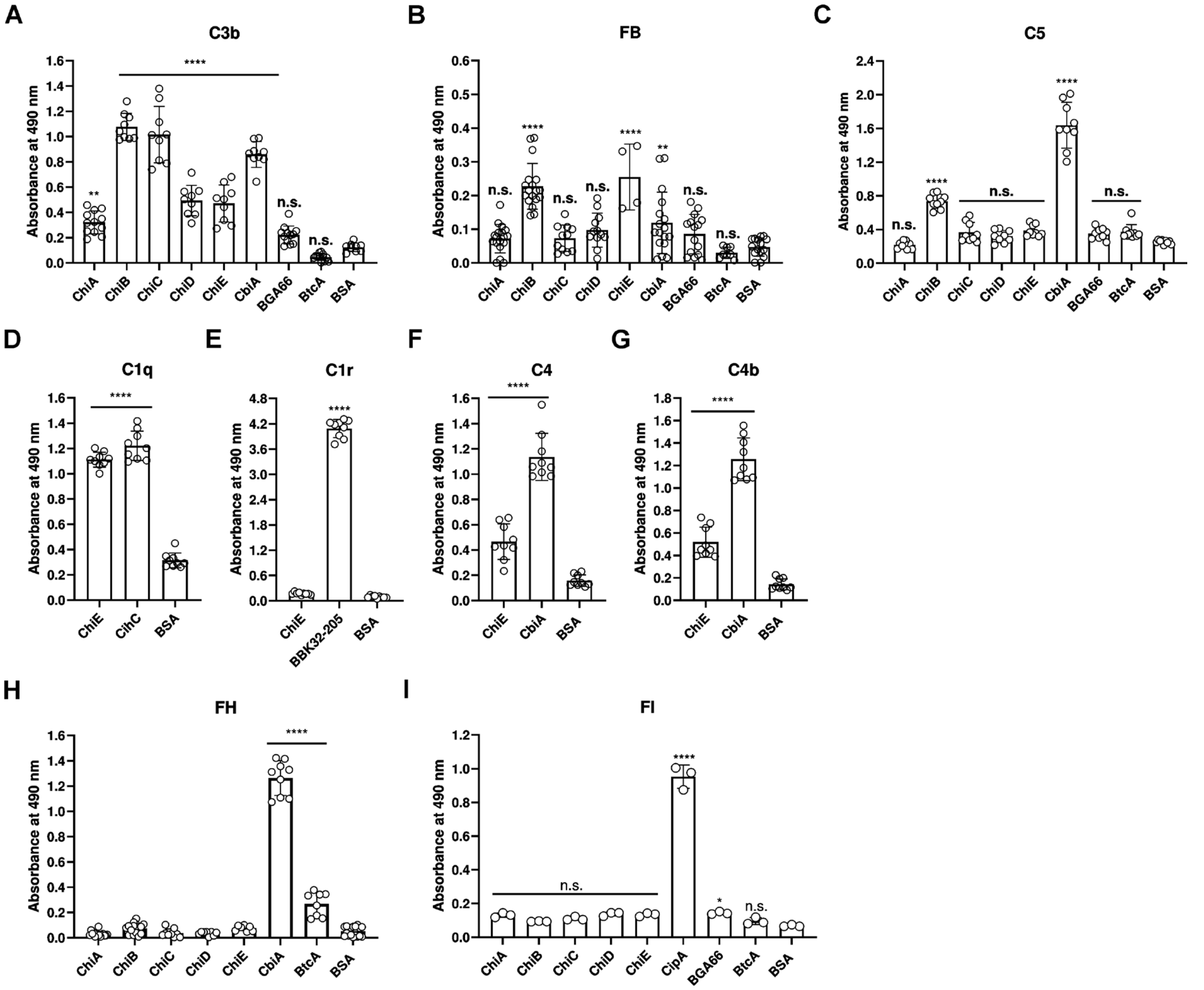

Binding of Chi proteins to different complement components of the AP, CP, and LP. Binding of C3b (A), FB (B), C5 (C), C1q (D), C1r (E), C4 (F), C4b (G), FH (H), and FI (I), respectively, to Chi proteins was measured by ELISA. Purified proteins (5 ng/µl) were immobilized and incubated with 5 ng/µl purified complement components. CbiA, BGA66, BtcA, ChiC, BBK32-205 were used as positive controls and BSA served as negative control. Bound complement components were detected using specific antisera (n=9, technical replicates, mean, ±SD). To assess statistical significance one-way ANOVA with Bonferroni post-hoc test (confidence interval = 95%) was performed. *, $p < 0.1$; **, $p < 0.01$; ***, $p < 0.001$; ****, $p \leq 0.0001$.

Supplementary figure 10

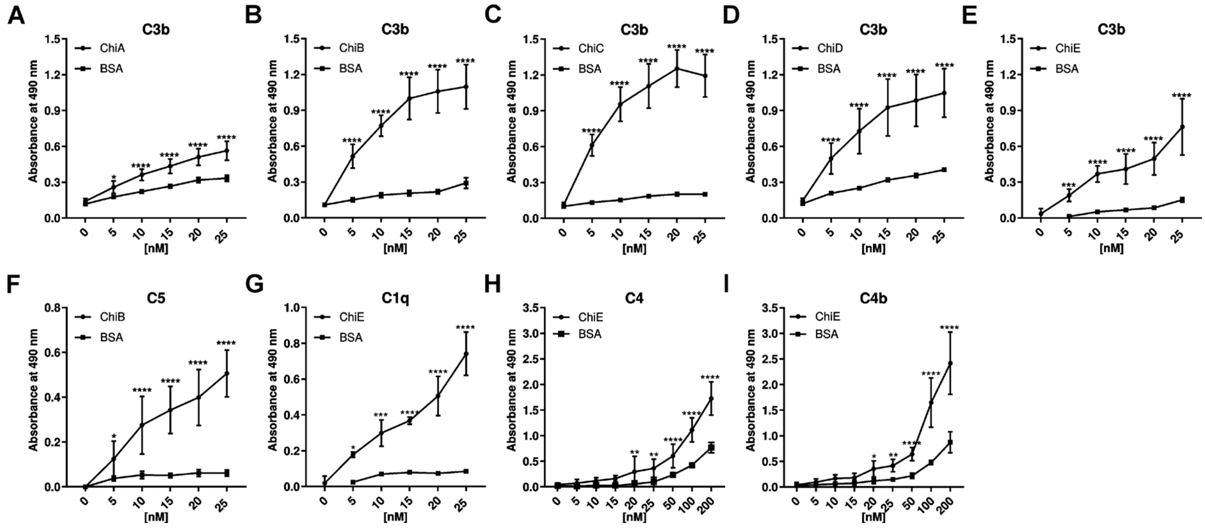

Dose-dependent binding of Chi proteins to different complement components. Binding of ChiA to C3b **(A)**, ChiB to C3b **(B)**, ChiC to C3b **(C)**, ChiD to C3b **(D)**, ChiE to C3b **(E)**, ChiB to C5 **(F)**, ChiE to C1q **(G)**, ChiE to C4 **(H)**, and ChiE to C4b **(I)**, respectively, was measured by ELISA. Chi proteins (5 ng/µl) were immobilized and incubated with increasing concentrations of different complement components as indicated. BSA (■) was used as negative control. Bound complement components were detected using specific antisera (1:1,000) (n=9, technical replicates, mean, ±SD). To assess statistical significance, one-way ANOVA with post-hoc Bonferroni multiple comparison test (confidence interval = 95%) was performed. Data represent means and/or standard deviation of at least three different experiments, each conducted in at least triplicate.*, $p \leq 0.1$; **, $p \leq 0.01$; ***, $p \leq 0.001$; ****, $p \leq 0.0001$.

Supplementary figure 11

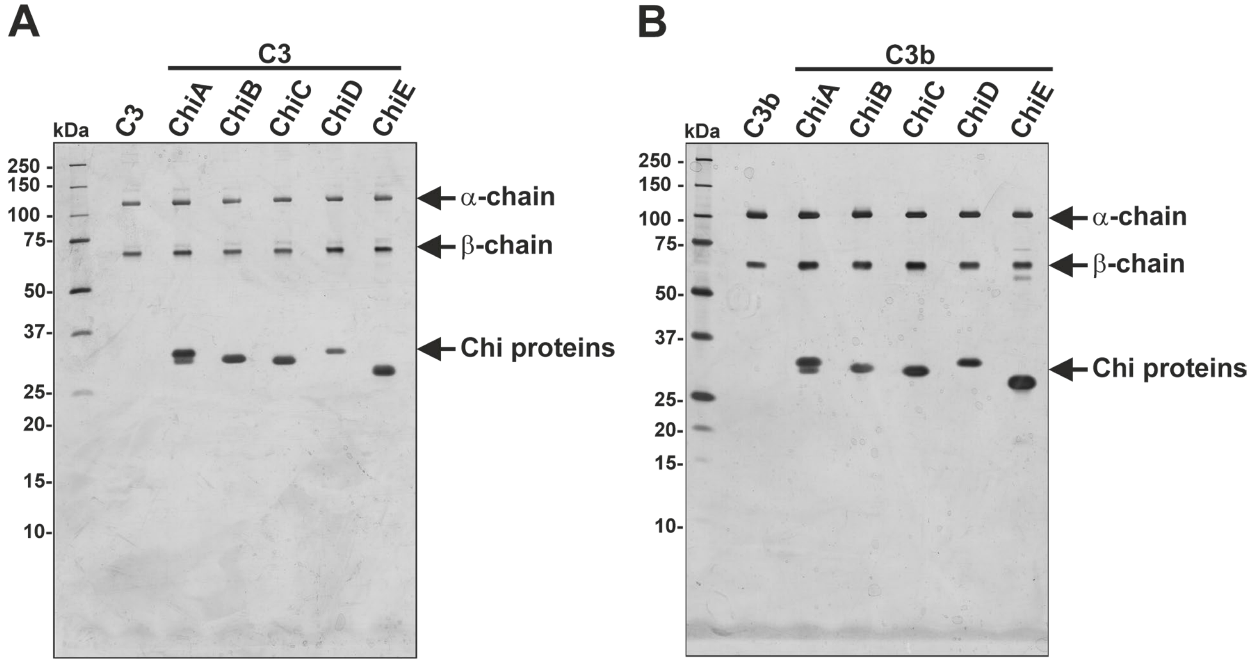

Determination of the proteolytic activity of Chi proteins. Chi proteins lack proteolytic activity to degrade complement C3 and C3b. Chi proteins (1 µg each) were incubated with complement C3 and C3b (500 ng each), respectively, for 2 h at 37 °C. Reaction mixtures were subjected to 10 % Tris/tricin SDS-PAGE and proteins were then visualized by silver staining. Purified C3 and C3b were used as controls. The mobilities of molecular mass standards (Precision Plus Protein Standards, Bio-Rad) are shown to the left of both panels

Supplementary figure 12

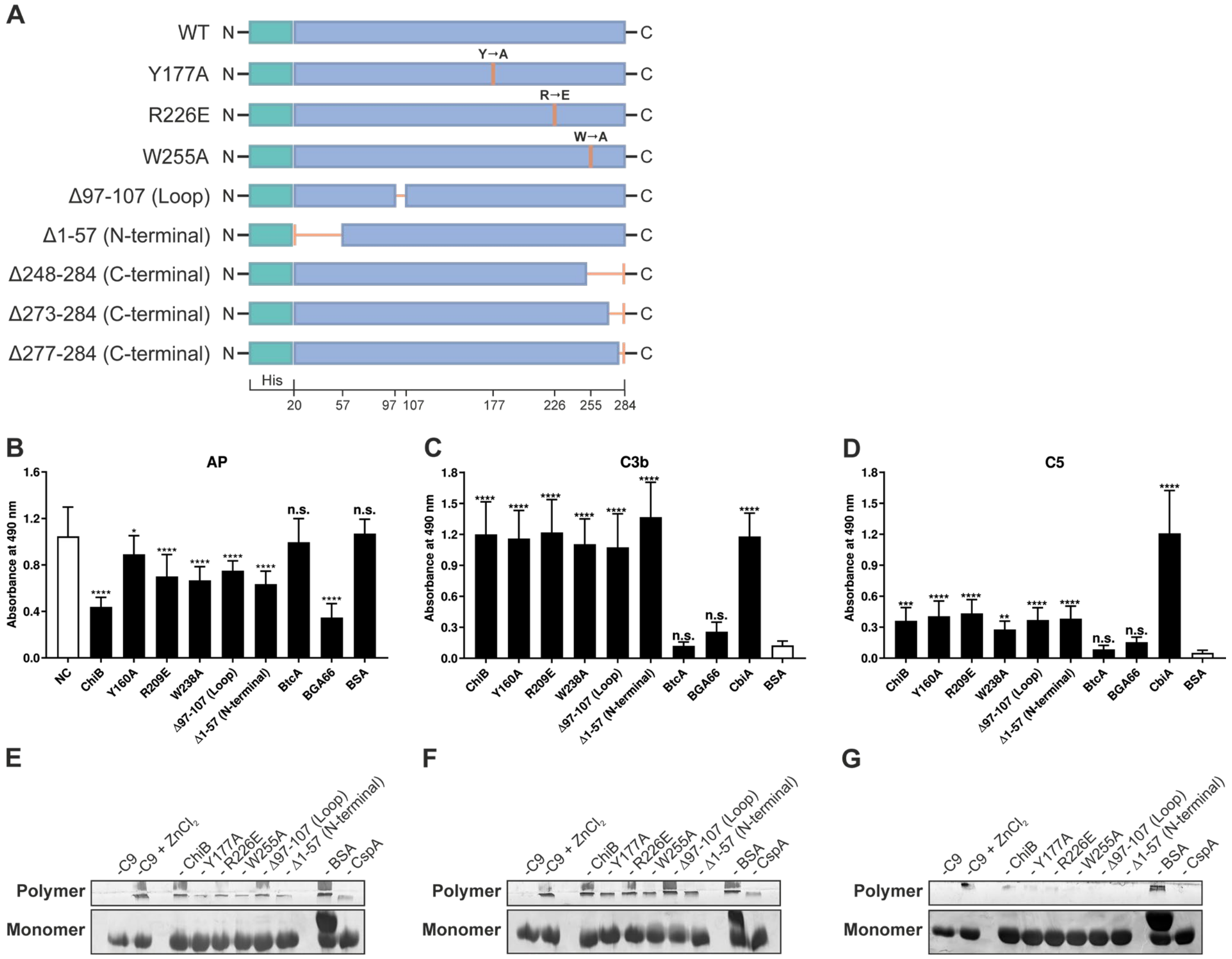

Functional analyses of ChiB variants. Schematic representation of ChiB variants carrying single and multiple deletions or substitutions **(A)**. Assessment of the inhibitory capacity of ChiB variants on activation of the AP

(B). NHS pre- incubated with the purified ChiB or BSA were added to microtiter plates immobilized with LPS. Formation of the MAC was detected by using a monoclonal anti-C5b-9 antibody. Binding of ChiB variants to C3b and C5 **(C and D)** (n=12, technical replicates, mean, ±SD). *, p ≤ 0.1; **, p ≤ 0.01; ***, p ≤ 0.001; ****, p ≤ 0.0001, n.s., no statistical significance, one-way ANOVA with post-hoc Bonferroni multiple comparison test (confidence interval = 95%). Inhibition of C9 polymerisation by ChiB variants **(E-G)**. C9 was pre-incubated with 2.0 µM **(E)**, 4.0 µM **(F)** or 7.5 µM **(G)** of purified ChiB variants, CspA (positive control) or BSA (negative control) and $ZnCl_2$ were then added to the reaction mixture to induced C9 autopolymerization. C9 incubated with and without $ZnCl_2$ was used as a further control. Following incubation, reactions mixtures were separated by 7.5% SDS-PAGE and C9 monomers and high molecular weight polymers were visualized by silver staining.

Supplementary figure 13

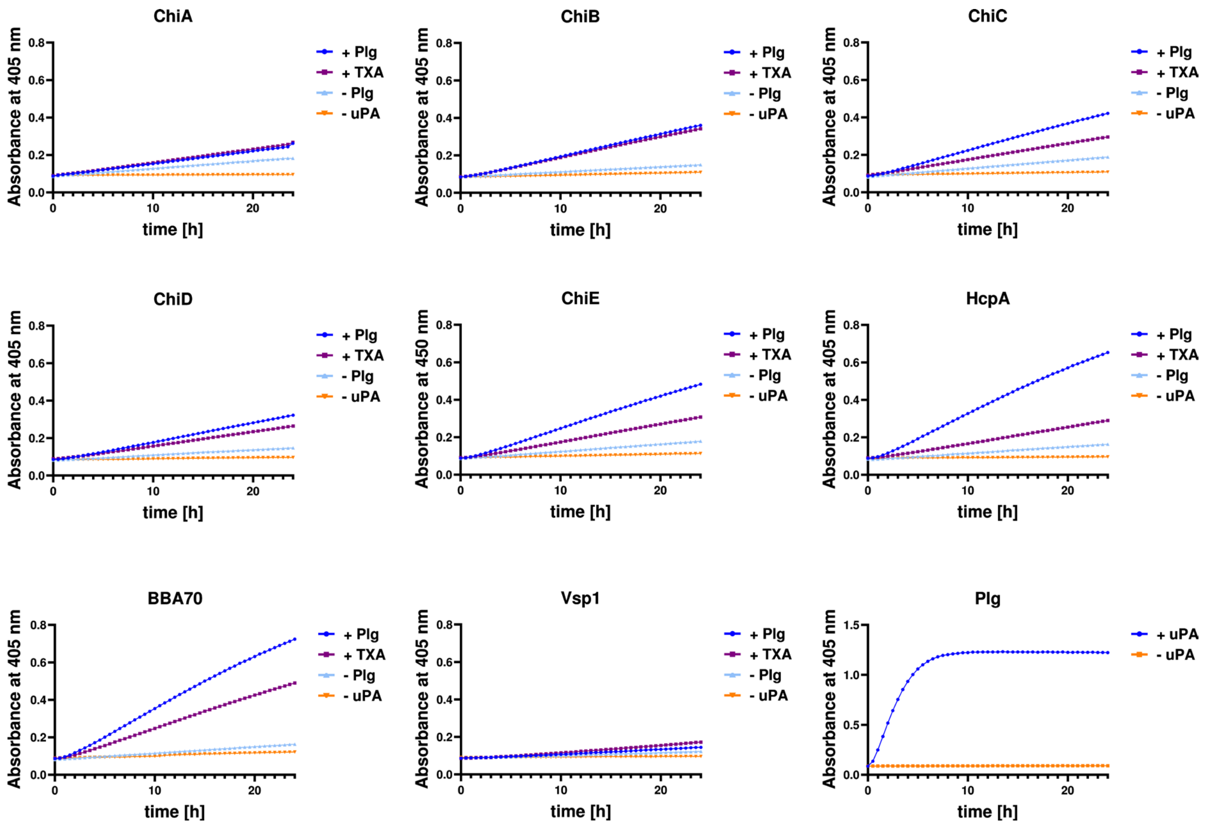

Plasminogen is converted to active plasmin upon binding to Chi proteins of *B. recurrentis*. Microtiter plates were coated with 5 µg/ml of Chi proteins, HcpA, BBA70, Vsp1 or plasminogen (Plg). The purified proteins were subsequently incubated with 10 µg/ml plasminogen. Following several wash steps, a reaction mixture containing the plasminogen activator uPA (final concentration of 0.1 µg/ml) and the chromogenic substrate D-Val-Leu-Lys-p-nitroanilide dihydrochloride (S-2251) was added (■). Control reactions included 50 mM of the lysine analog tranexamic acid (T) (♦) or omitted plasminogen (▼) or uPA (▲), respectively. Microtiter plates were incubated at room temperature for 24 h and absorbance at 405 nm was measured at 30 min intervals (n=9, technical replicates, mean, ±SD).

Supplementary figure 14

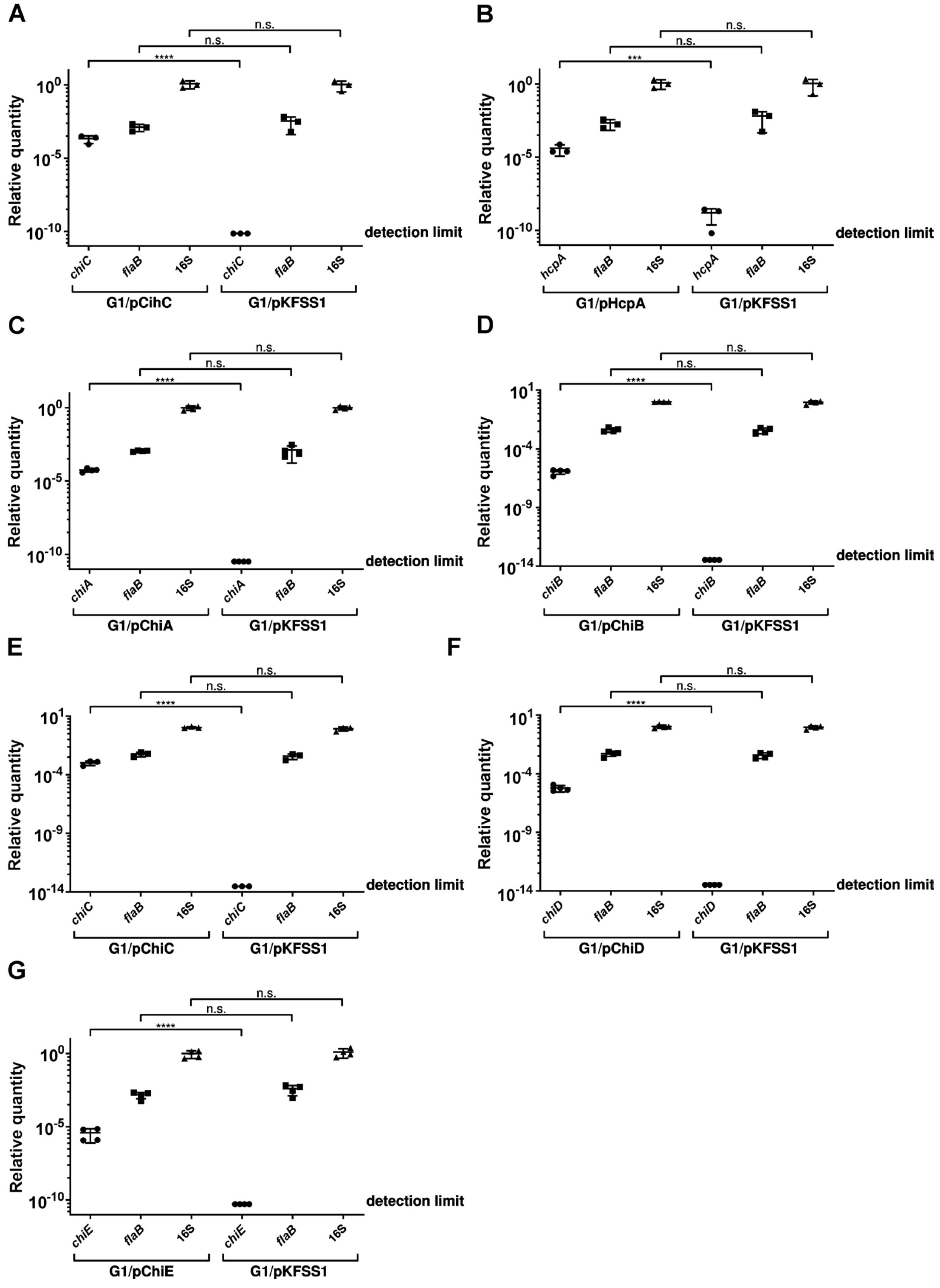

Gene expression analyses of Chi-encoded genes in gain-of-function strains. Expression of chi homologues genes in in vitro cultivated gain-of-function strains was determined by quantitative real-time PCR analyses. Total RNA was isolated from in vitro gain-of-function strains grown at 33 °C and transcribed to cDNA. Differences were calculated by comparing the CT values with those obtained from transcribed cDNA of the serum-sensitive *B. garinii* G1 strain carrying the empty shuttle vector G1/pKFSS1 (n=3, biological replicates, mean, ±SD). Differences were calculated by the $2^{-\Delta\Delta CT}$ method. An unpaired student t-test with a confidence interval of 95% was used to calculate the statistical significance. ***, $p \leq 0.001$; ****, $p \leq 0.0001$, n.s., no statistical significance.

Supplementary figure 15

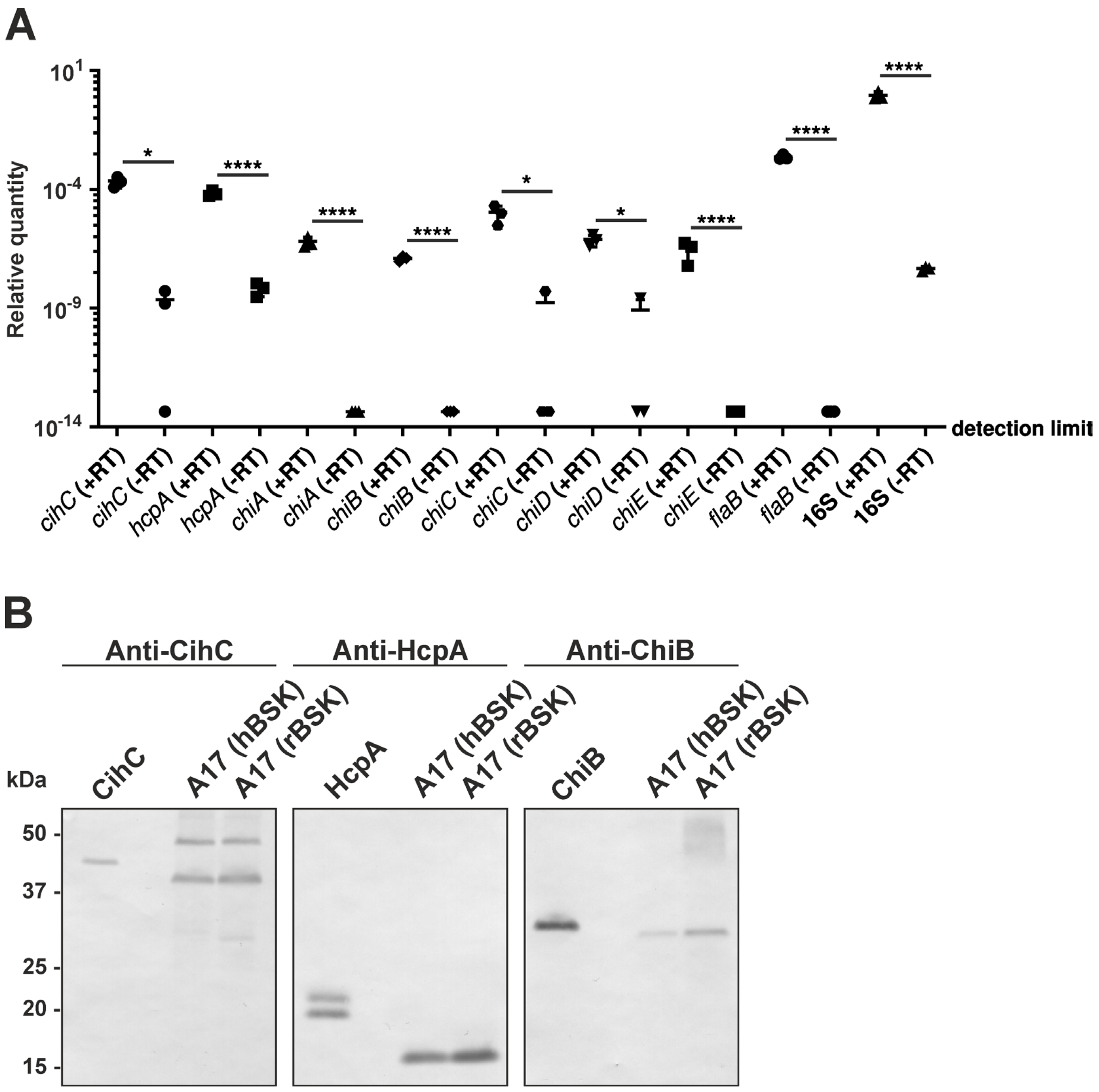

Gene expression of Chi-encoded genes and detection of selected complement-targeting proteins in *B. recurrentis* PAbJ. Gene expression of different Chi-encoded genes in *in vitro* cultivated *B. recurrentis* PAbJ was determined by quantitative real-time PCR analyses **(A)**. Total RNA was isolated from *in vitro* spirochetes grown at 33 °C and transcribed to cDNA (+RT). Average C_T values were normalized to reactions lacking reverse transcriptase (-RT) and differences were calculated using the $2^{-\Delta\Delta CT}$ method (n=3, biological replicates, mean, ±SD). Significant differences were calculated by the unpaired student t-test with a confidence interval of 95%. *, p ≤ 0.1; **, p ≤ 0.01, ***, p ≤ 0.001; ****, p ≤ 0.0001; n.s., no statistical significance. Detection of ChiC, HcpA, and ChiB in *in vitro* grown *B. recurrentis* PAbJ by Western blot analyses **(B)**. Purified proteins (500 ng each) as well as cell lysates (20 µg each) prepared from cultures grown in BSK medium supplemented with human serum (hBSK) or with rabbit serum (rBSK) were separated by 10% Tris/tricine SDS-PAGE and transferred to nitrocellulose. Proteins were detected by specific mAb directed to CihC (Grosskinsky et al., 2010) and HcpA (Schott et al., 2009), respectively or ChiB applying a polyclonal rabbit anti-ChiB antibody.

Supplementary figure 16

A. Potential canonical promoter elements within the 5´upstream region of the *chiA* gene

5´taaaagatgtttaaataaatgatataaaaagctaataaaatatattgatatctaggtcaaattaatattaactatatgttaataatgattatta
att aatatacagcaaacaatttgaataaattgttttattttaataataaggagagtatttttg- 3´

Core Promoter Element Matches				
**Motif**	**Position**	**Score**	**Sequence**	**TSS**
INR	89	0.97	ccagttt	91
TATA	18	0.98	gatataaaagc	47
TATA	9	0.89	taaataaatgat	38
TATA	113	0.88	tgaataaattgt	142
TATA	29	0.88	ctaataaaatat	58
TATA	5	0.85	tgtttaaataaa	34
TATA	16	0.83	atgatataaaaa	45
TATA	34	0.81	aaaatatattga	63
**Synergistic Combination Matches**				
TATA + INR	18 + 54	1.82	gatataaaagc + ccagttt	52
TATA + INR	29 + 54	1.72	ctaataaaatat + ccagttt	52
TATA + INR	34 + 54	1.65	aaaatatattga + ccagttt	52

B. Potential canonical promoter elements within the 5´upstream region of the *chiB* gene

5´tagtaagaatttatgttgattatgaatcaattaaatatatttataaatactattggattaaacatgactgaggaaaagttttccttggtgatcg
ata attagtcagtttatcaattg-3´

Core Promoter Element Matches				
**Motif**	**Position**	**Score**	**Sequence**	**TSS**
INR	100	0.95	tcagttt	102
TATA	37	0.94	tttataaatact	66
TATA	29	0.81	taaatatattta	58
DPE	98	0.91	agtca	70
**Synergistic Combination Matches**				
TATA + DPE	37 + 98	1.85	tttataaatact + agtca	70

C. Potential canonical promoter elements within the 5´upstream region of the *chiC* gene

5´taaaaggttaattttgattattaatcaatttaaaaatactattaattaaatataaccaagaaaaattttccttggttatcaataattagttagtttata attagataatgaggagaatatttttg-3´

Core Promoter Element Matches				
**Motif**	**Position**	**Score**	**Sequence**	**TSS**
INR	22	0.94	tcaattt	24
INR	87	0.82	ttagttt	89
INR	5	0.82	ttaattt	7
TATA	24	0.85	aatttaaaaata	53
TATA	44	0.84	taaatataacca	73
DPE	100	0.93	agata	72
DPE	85	0.88	agtta	57
DPE	71	0.87	ggtta	43
**Synergistic Combination Matches**				
TATA + DPE	24 + 85	1.73	aatttaaaaata + agtta	57
TATA + DPE	44 + 100	1.76	taaatataacca + agata	72

D. Potential canonical promoter elements within the 5´upstream region of the *chiD* gene

5´taatgttaattataaatcaattaaatatatttaggacacttttaaattaaatataaccaaggaaaaatttttcttggttatatttaattagccagtttg taatttaatgataattgaataataaggaggatattttga-3´

Core Promoter Element Matches				
**Motif**	**Position**	**Score**	**Sequence**	**TSS**
INR	89	0.97	ccagttt	91
TATA	6	0.92	attataaatcaa	35
TATA	45	0.84	taaatataacca	74
TATA	19	0.81	taaatatattta	48
DPE	31	0.95	ggaca	3
DPE	125	0.92	ggaca	97
DPE	73	0.87	ggtta	45
**Synergistic Combination Matches**				
TATA + DPE	6 + 73	1.73	attataaatcaa + ggtta	45
TATA + DPE	19 + 73	1.76	taaatatattta + ggtta	45

E. Potential canonical promoter elements within the 5´upstream region of the *chiE* gene

5´taggagattaatgttaattataaatcaattaaatatatttaaaacactgttaaattaaatataaacaaggtaagaaattacttggttatcagtaatt agccagtttgtaattagataataaggaagatatttttg-3´

Core Promoter Element Matches				
**Motif**	**Position**	**Score**	**Sequence**	**TSS**
INR	97	0.97	ccagttt	99
INR	75	0.80	ttacttg	77
TATA	14	0.92	attataaatcaa	43
TATA	33	0.85	tatttaaaacac	62
TATA	53	0.85	taaatataaaca	82
TATA	27	0.81	taaatatattta	56
DPE	110	0.93	agata	82
DPE	122	0.93	agata	94
DPE	81	0.87	ggtta	53
**Synergistic Combination Matches**				

TATA + DPE	14 + 81	1.79	attataaatcaa + ggtta	53
TATA + DPE	27 + 81	1.68	taaatatattta + ggtta	53
TATA + DPE	33 + 81	1.72	tatttaaaacac + ggtta	53
TATA + DPE	53 + 110	1.78	taaatataaaca + agata	82
TATA + INR	53 + 75	1.65	taaatataaaca + ttacttg	73

F. Promoter element analyses of *chi* genes using Promotech

ChiA gene:

	chrom	start	end	score	strand	sequence
0	sequenz-promotech-ChiA	8	47	0.62794	+	GTTTAAATAAATGATATAAAAAGCTAATAAAATATATTGA
1	sequenz-promotech-ChiA	11	50	0.64037	-	ATATCAATATATTTTATTAGCTTTTTATATCATTTATTTA
2	sequenz-promotech-ChiA	57	96	0.64770	-	AATTAATAATCATTATTAACATATAGTTAATATTAATTTG

ChiB gene:

	chrom	start	end	score	strand	sequence
0	sequenz-promotech-ChiB	11	50	0.61688	-	AGTATTTATAAATATATTTAATTGATTCATAATCAACATA

ChiC gene:

	chrom	start	end	score	strand	sequence
0	sequenz-promotech-ChiC	67	106	0.65374	+	TTCCTTGGTTATCAATAATTAGTTAGTTTATAATTAGATA
1	sequenz-promotech-ChiC	68	107	0.71418	+	TCCTTGGTTATCAATAATTAGTTAGTTTATAATTAGATAA

ChiD gene:

	chrom	start	end	score	strand	sequence
0	sequenz-promotech-ChiD	0	39	0.68232	-	AGTGTCCTAAATATATTTAATTGATTTATAATTAACATTA
1	sequenz-promotech-ChiD	1	40	0.69834	-	AAGTGTCCTAAATATATTTAATTGATTTATAATTAACATT
2	sequenz-promotech-ChiD	42	81	0.72711	-	TATAACCAAGAAAAATTTTTCCTTGGTTATATTTAATTTA

ChiE gene:

	chrom	start	end	score	strand	sequence
0	sequenz-promotech-ChiE	6	45	0.66549	+	ATTAATGTTAATTATAAATCAATTAAATATATTTAAAACA
1	sequenz-promotech-ChiE	8	47	0.70451	-	AGTGTTTTAAATATATTTAATTGATTTATAATTAACATTA
2	sequenz-promotech-ChiE	9	48	0.68325	-	CAGTGTTTTAAATATATTTAATTGATTTATAATTAACATT
3	sequenz-promotech-ChiE	23	62	0.74323	+	ATCAATTAAATATATTTAAAACACTGTTAAATTAAATATA
4	sequenz-promotech-ChiE	50	89	0.63862	-	GATAACCAAGTAATTTCTTACCTTGTTTATATTTAATTTA

Prediction of potential canonical promoter motifs within the 5´upstream region of the five *chi* genes of *B. recurrentis* A17. The YAPP Eukaryotic Core Promoter Predictor **(A-E)** was used to search for TATA boxes, initiator elements (INR), downstream core element (DPE), and for putative synergistic combinations (https://www.bioinformatics.org). The YAPP tool is created for scanning upstream sequences of putative determined promoter regions in eukaryotic but also for prokaryotic promoter sequences. Perfect matches receives a score of 1.0 and matrix similarity score >0.8 is considered good and, therefore, the optimum cutoff value in the analysis. The stop codon is highlighted in yellow and start codon in light blue. TSS, transcription start site. Promotech **(F)**, a machine-learning-based method, was used as a separate tool to recognize promoter regions within the upstream region of each individual *chi* gene (https://github.com/BioinformaticsLabAtMUN/PromoTech) and developed by R. Chevez-Guardado & L. Peña-Castillo, 2021 (https://doi.org/10.1186/s13059-021-02514-9). True positives predictions display score values that tend to be around 0.5 or higher.

Supplementary figure 17

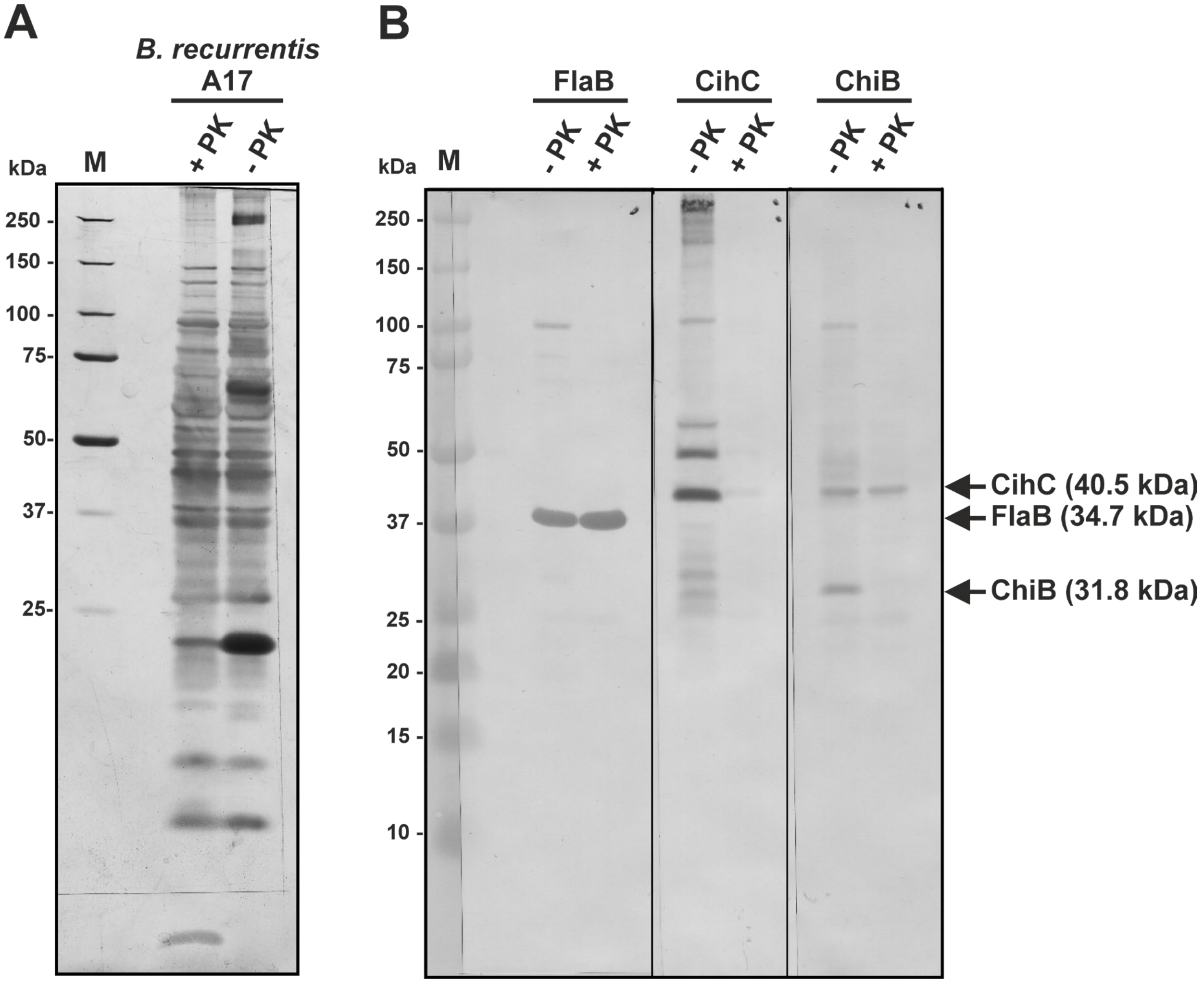

Surface exposition of CihC and ChiB. After *in situ* treatment of native *B. recurrentis* A17 with or without proteinase K, cell lysates (20 µg) were subjected to 10% TT-SDS-PAGE and proteins were visualized by silver staining **(A)** and Western blotting **(B)**. CihC and ChiB were analyzed by a monoclonal anti-CihC antibody (Grosskinsky et al., 2010) (1:10) and a polyclonal anti-ChiB antibody (1:1000), respectively. As a control, the periplasmatic FlaB protein was investigated applying a monoclonal anti-FlaB Ab L41 1C11 (1:100) (Hauser et al., 1999)[66]. The proteins corresponding to CihC, ChiB, and FlaB, respectively are indicated on the right. The mobilities of molecular mass standards (Precision Plus Protein Standards, Bio-Rad) are indicated on the left.

Supplementary figure 18

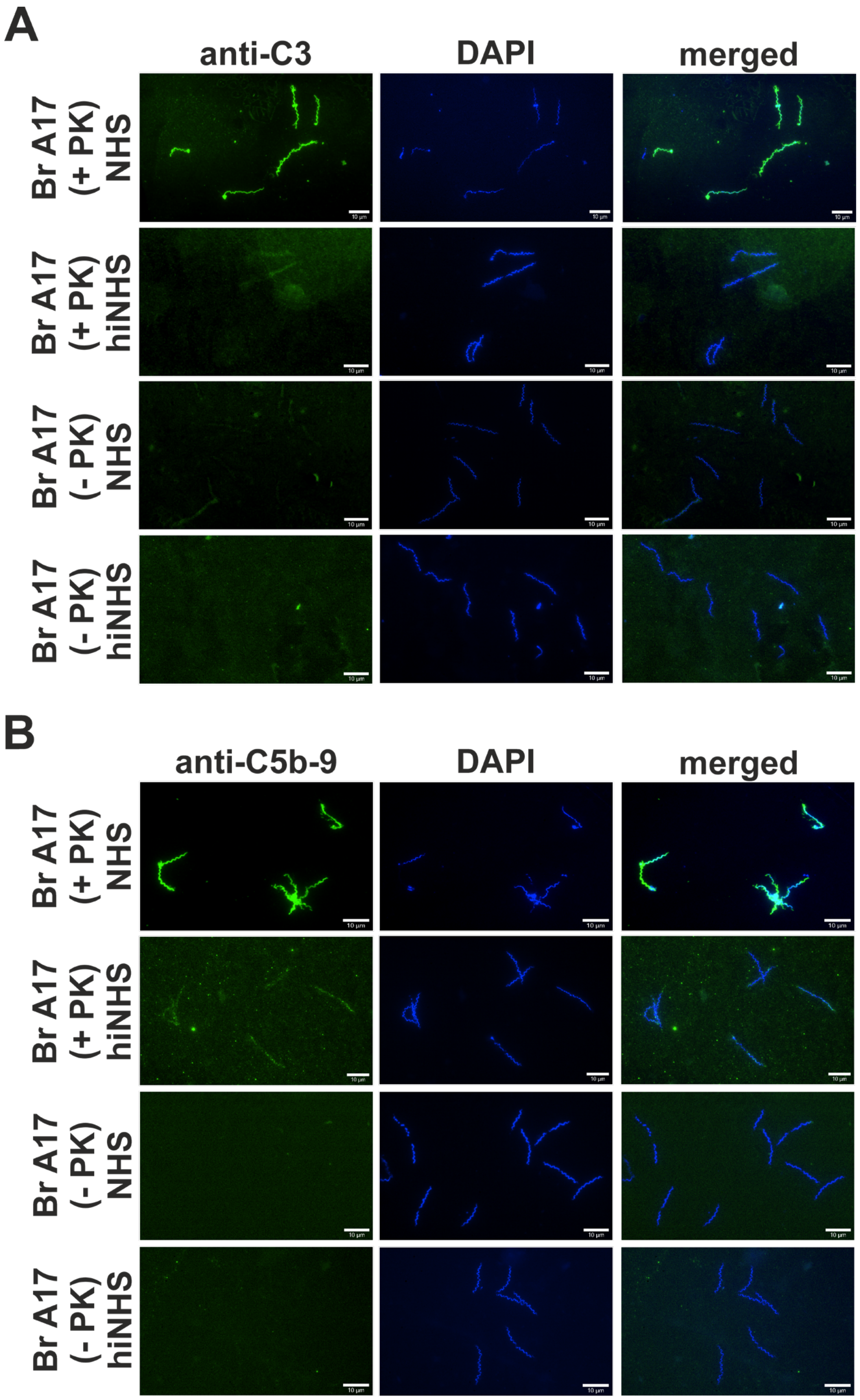

Deposition of activated complement components C3 and C5b-9 (MAC) on the surface of proteinase K-treated *B. recurrentis* A17. Complement activation on proteinase K-treated and untreated *B. recurrentis* A17 (6×10^6 cells/µl) were detected after incubation of spirochetes with NHS or hiNHS. After fixation, deposition of C3 **(A)** and the formed MAC **(B)** were visualized applying a polyclonal anti-C3 Ab (1:1000) or a monoclonal anti-C5b-9 Ab (1:50). Spirochetal DNA (blue) was stained with DAPI. All scale bars are equal to 10 µm. Spirochetes were observed at a magnification of 1,000 and the data were recorded with an Axio Imager M2 fluorescence microscope (Zeiss) equipped with a Spot RT3 camera (Visitron Systems). All images shown are representative.

Supplementary Table 1. Amino acid identity and similarity of Chi orthologous proteins of *B. recurrentis* A1.

	ChiA	ChiB	ChiC	ChiD	ChiE	
ChiA	100	66.9	64.8	64.8	76.1	Similarity (%)
ChiB	38.0	100	70.2	73.1	60.1	
ChiC	38.4	44.3	100	75.3	63.1	
ChiD	39.4	48.3	56.5	100	63.4	
ChiE	48.7	33.7	36.2	40.3	100	
	Identity (%)					

The matrix was generated using Lalign with following settings: Smith-Waterman algorithm, BLOSUM50, opening gap penalty: -12, extending gap penalty: -2, threshold score: 10.0. NCBI accession numbers used were as follows: WP_376984189 (ChiA), WP_376984193 (ChiB), WP_376984195 (ChiC), WP_376984196 (ChiD), WP_376984198 (ChiE).

Supplementary Table 2. Data collection and refinement statistics

	ChiA	ChiB
PDB code	28LI	28LK
**Data collection**		
Space group	C 1 2 1	P 42 21 2
Cell dimensions		
a, b, c (Å)	53.07 115.84 83.03	82.01 82.01 90.42
α, β, γ (°)	90 90.032 90	90 90 90
Resolution (Å)	48.3-2.7 (2.8-2.7)	36.7-1.5 (1.55-1.5)
R-merge (%)	8.5 (86.5)	8.5 (251.9)
$I/\sigma I$	13.5 (2.1)	13.7 (0.9)
Completeness (%)	98.2 (94.7)	99.8 (99.1)
Redundancy	6.5 (5.8)	10.6 (10.5)
Molecules per AU	2	1
Wilson B-factor	63.5	23.8
$CC_{1/2}$ (%)	99.9 (80.2)	99.9 (35.2)
**Refinement**		
Resolution (Å)	2.7	1.5
Unique reflections	13528 (1302)	49770 (4840)
R_{work}/R_{free} (%)	25.3(33.9)/30.1(38.8)	18.1(31)/21.5(35.2)
No. atoms		
Proteins	3653	2048
Ligands	2	62
Water	15	263
Protein residues	461	256
B-factors		
Proteins	72.8	31.7
Ligands	56.1	48.9
Water	62.1	43.9
Ramachandran plot (%)		
Favored (%)	97.1	99.2
R. m. s. deviations		
Bonds lengths	0.004	0.014
Bond angles	0.97	0.94

Values in parentheses are for highest-resolution shell.

Supplementary Table 3. Data collected by the DTNB assay

protein	#cysteine	date	red with	red	exp	date + X	ox	exp
ChiA	C115 (pocket)	05/02/2025	DDT	0.83	1	5	0.20	0
	C260 (surface)			0.83				
		11/02/2025	DDT	0.97	1	6	0.37	0
	S-S: 260-260’			0.90			0.40	
		13/02/2025	BME	0.23	1	5	0.03	0
				0.23			0.03	
		mean +/- s		0.66 +/-0.18	1		0.21 +/-0.15	0
ChiB	C266 (pocket)	05/02/2025	DDT	0.23		5	0.2	
				0.37				
		11/02/2025	DDT	0.11		6	0.02	
				0.11			0.02	
		mean +/- s		0.21 +/-0.12	0-1		0.08 +/-0.13	0-1
ChiC	C227 VR-loop	05/02/2025	DDT	1.7	3	5	1.17	1
				1.8				
	C165 (pocket)	27/02/2025				21	0.96	
	C260 (pocket)	11/02/2025	DDT	1.25	3	6	0.99	1
				1.34			0.99	
	S-S: C165-C260	27/02/2025				15	1.01	
		13/02/2025	BME	1.29	3	5	1.22	
				1.34			1.21	1
		27/02/2025				8	0.66	
		mean +/- s		1.45 +/- 0.23	3		1.03 +/-0.18	1
ChiD	C264 (pocket)	05/02/2025	DDT	0.85		5	0.59	
	acc.			0.80				
	protected by	11/02/2025	DDT	0.47		6	0.23	
	K166 & N265	13/02/2025	BME	0.34				
				0.31		5	0.51	
							0.17	
		mean +/- s		0.55 +/-0.24	0-1		0.375 +/- 0.1	0-1
ChiE	C156 (pocket)	05/02/2025	DDT	2.48	2	5	0.47	0
	C227 (pocket)			1.98				
	S-S: C156-C227	11/02/2025	DDT	1.71	2	6	0.70	0
				1.72				
		13/02/2025	BME	1.86	2	5	0.77	0
				2.52			0.81	
		mean +/- s		2.05 +/- 0.31	2		0.57 +/- 0.1	0

Supplementary Table 4. Functional characterization of Chi proteins

	ChiA	ChiB	ChiC	ChiD	ChiE
AP Inhibition	yes	yes	yes	yes	yes
CP Inhibition	no	no	no	no	yes
LP Inhibition	no	no	no	no	yes
TP Inhibition	no	yes	yes	yes	yes
Binding of complement components	C3b	C3b, C5, C9	C3b, C9	C3b, C9	C1q, C3b, C4, C4b, C9
Inhibition of C9-polymerization	no	yes	yes	yes	yes
Binding of plasmin(ogen)	yes	yes	yes	yes	yes
Involvement of lysines	no	no	yes	no	yes
Gene expression in *B. recurrentis*	yes	yes	yes	yes	yes
Protection of sensitive spirochetes	no	yes	yes	yes	yes
Confers serum resistance (gain-of-function strains)	no	yes	yes	yes	no

AP, alternative pathway, CP, classical pathway, LP, lectin pathway, TP, terminal pathway

Supplementary Table 5. Oligonucleotides used in this study

Oligonucleotide	Sequence (5'-3')[a]	Use in this work
**Generation of ChiB variants with single aa substitutions, N-terminal and internal deletions**		
ChiB_Y177A FP	GATTTTGCTAAAGCTGCTTATATAGATGCACATGATACCTTAC	Generation of variant ChiBY177A
ChiB_Y177A RP	GTATCATGTGCATCTATATAAGCAGCTTTAGCAAAATCTC	Generation of variant ChiBY177A
ChiB_R226E FP	GGTGTAATAGCCGAAGTTGAAAGTGATTTAAATAAC	Generation of variant ChiBR226E
ChiB_R226E RP	CTACTGTTATTTAAATCACTTTCAACTTCGGCTATTAC	Generation of variant ChiBR226E
ChiB_W255A FP	GCAGATAAAAACATATGCGAATCAAAGTCAGCTAC	Generation of variant ChiBW255A
ChiB_W255A RP	GCTGACTTTGATTCCGATATGTTTTTATCTGCTCCG	Generation of variant ChiBW255A
ChiB_Δ1-57 FP	GAAAAAGAGAAATTGGATCCATTAAAGATGCAACTTCTAG	Generation of variant ChiBΔ1-57 lacking N-terminal aa 1-57
ChiB_Δ97-107 FP	GGTGTAACTGAAGTCGTGCACAATGATGCAGCTG	Generation of variant ChiBΔ87-107 lacking the loop region aa 97-107
ChiB_Δ97-107 RP	CCATCTACTTTATGGTGCACTTTACCAAACAC	Generation of variant ChiBΔ87-107 lacking the loop region aa 97-107
ChiB_P248_Stop RP	CAATATGTTTTTATCTGGTCGACGGTGGTAGCATTAC	Generation of variant ChiBΔP248-Q284 lacking the C-terminal aa 248 to 284
ChiB_S273_Stop RP	CAAAATATTTTTAATTTAACCTCATATCTTCACAATCTCAG	Generation of variant ChiBΔS273-Q284 lacking the C-terminal aa 273 to 284
ChiB_K277_Stop RP	CTGAATATTATCCAAAATATTTTAAATTTCACCACTTATCTTC	Generation of variant ChiBΔK277-Q284 lacking the C-terminal aa 277 to 284
**Generation of shuttle vectors**		
ORF5_Cterm_FP_SacI	GAAAATTAAAAATATTAAAGAGCTCAGTAATGATATTAAAG	Generation of shuttle vector pChiA
pChiB_Nterm_RP_SalI	GTCTTTCCTTTTTTGCTTGGTCGACTTTTTGTTGTGCC	Generation of shuttle vector pChiA
pChiA_Cterm_FP_SacI	GGAACAAAAAGAGCTCTGGTTTTGAAACTCG	Generation of shuttle vector pChiB
pChiC_Nterm_RP_SalI	CCTCTCCTCTTCTTCTGATTGTCGACGTGACGATGC	Generation of shuttle vector pChiB
pChiB_Cterm_FP_SacI	GATGACTGAGCTCAGATTGTGAAGATAAGTGG	Generation of shuttle vector pChiC
pChiD_Nterm_RP_v2_SalI	CAGGCTTTTTTCCGTGTCGACCACAAGC	Generation of shuttle vector pChiC
pChiC_Cterm_FP_SacI	GAGGATAAGAGCTCAAATTAAAGGAATCTTGG	Generation of shuttle vector pChiD
pChiE_Nterm_RP_v2_SalI	GTGTCTCTGCATCATAAAGTCGACTTTCTCC	Generation of shuttle vector pChiD
pChiD_Cterm_FP_SacI	GAGATCTTGACTAGAGCTCATTAGGAGATTAATG	Generation of shuttle vector pChiE
ORF11_Nterm_RP_SalI	GATCACAAGCTATTAGTAGTCGACTTATCAACAC	Generation of shuttle vector pChiE
**Primer used for RT-qPCR**		
CihC FP RT	ATATTACTAGCAGTTTAAGCGGT	RT-qPCR, amplification of *cihC*
CihC RP RT	GGACTATCTTCTTCTTCCACAATAC	RT-qPCR, amplification of *cihC*
HcpA FP RT	TTTCAAGAATCACTTCAAGATAATAAGC	RT-qPCR, amplification of *hcpA*
HcpA RP RT	AGTCTTATCATTTCCAAGTTTCCT	RT-qPCR, amplification of *hcpA*
ChiA FP RT	GGTACAACGCCTAATTCT	RT-qPCR, amplification of *chiA*
ChiA RP RT	CCAGATCTTCCAATCTTCC	RT-qPCR, amplification of *chiA*
ChiB FP RT	TGCGACTGGCGCTGTTG	RT-qPCR, amplification of *chiB*
ChiB RP RT	CAAAAATCTTGCCTCGTCTAAAGAGAG	RT-qPCR, amplification of *chiB*
ChiC FP RT	ACAAAGTCTAGCGATAGG	RT-qPCR, amplification of *chiC*
ChiC RP RT	GTAACTAAGTCCCTAGTCTC	RT-qPCR, amplification of *chiC*
ChiD FP RT	CTAATGGATGACAATAGGGC	RT-qPCR, amplification of *chiD*
ChiD RP RT	TAAGTCCACAGTCCCAAT	RT-qPCR, amplification of *chiD*
ChiE FP RT	GGTACGACACTTGGCGAT	RT-qPCR, amplification of *chiE*
ChiE RP RT	CGATTCTAAAATCCCTAATGAATCAGAACC	RT-qPCR, amplification of *chiE*
FlaB FP RT	CTAGTGGGCATAGAATTAATCGTGC	RT-qPCR, amplification of *flaB*
FlaB RP RT	GCTTGGGATAACCCTCTAATTTGA	RT-qPCR, amplification of *flaB*

FlaB *B. garinii* FP RT	ATCAAACAAATCTGCTTCCCA	RT-qPCR, amplification of *flaB* (*B. garinii* G1)
FlaB *B. garinii* RP RT	ATATTTACAGCAATCGCTTCATC	RT-qPCR, amplification of *flaB* (*B. garinii* G1)
16S FP RT	GCTGTAAACGATGCACACTTGGT	RT-qPCR, amplification of 16S rRNA
16S RT RT	GGCGGCACACTTAACACGTTAG	RT-qPCR, amplification of 16S rRNA
**Primer used for cloning of the BDU1066 encoding gene of *Borrelia duttonii* Ly**		
Bre_1066_FP_Bam	GGAGTAATATGGATCCGAAATGAAATCTTCTTTTGG	Cloning of the Bdu1066 encoding gene
Bre_1066_RP_Sal	CCTAAATTTATAACATCAGTCGACTTATAAAAGATCAACC	Cloning of the Bdu1066 encoding gene
**Primer used for re-cloning of the BBK32 encoding gene of *Borrelia burgdorferi* B31 and generation of a C-terminal BBK32 fragment**		
BBK32 Bam_FP	GGAACTCGGGATCCGATTTATTCATAAGATAT	Recloning of the BBK32 encoding gene in pQE-30 Xa
BBK32 Hind_RP	GCAAGCTTTAAGTACCAAACGCCATTCTT	Recloning of the BBK32 encoding gene in pQE-30 Xa
BBK32-205 BamHI	GAGGATGAAGAGGGATCCAGATTAAGCAATCGATATC	Generation of the C-terminal BBK32 fragment BBK32205
**Primer used for sequencing**		
pQE-FP-30	TTGCTTTGTGAGCGGATAAC	Sequencing of inserted DNA fragments cloned into pQE-30 Xa
pQE-RP	CTGAGGTCATTACTGGATCTATC	Sequencing of inserted DNA fragments cloned into pQE-30 Xa
M13 FP	GTAAAACGACGGCCAGT	Sequencing of inserted DNA fragments cloned into shuttle vector pKFFS1
M13 RP	CAGGAAACAGCTATGAC	Sequencing of inserted DNA fragments cloned into shuttle vector pKFFS1

[a], Sequences of specific restriction endonuclease recognition sites are underlined

Supplementary Figures – Uncropped blots and gels

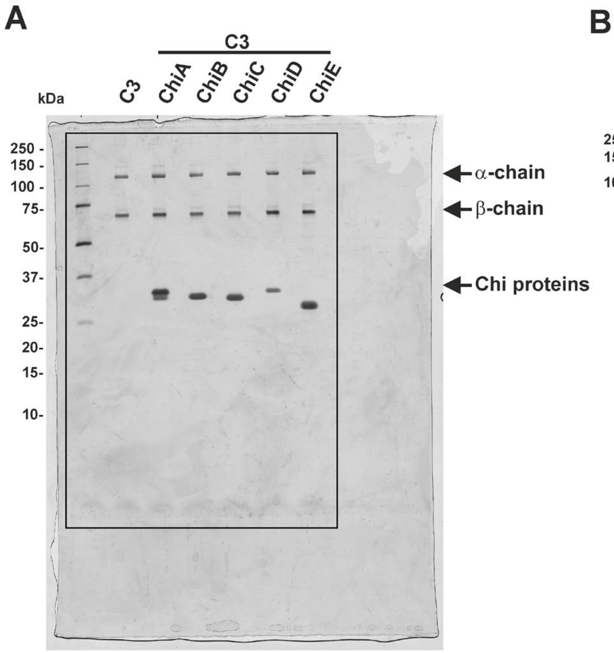

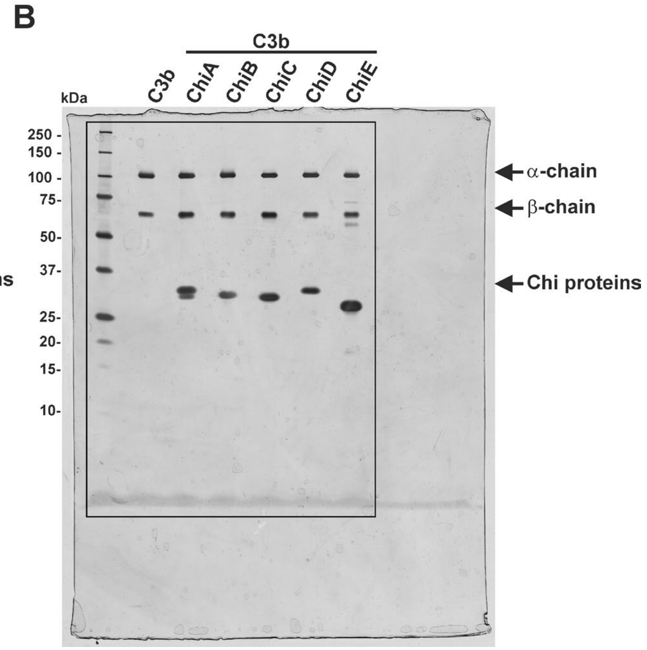

Supplementary Figure S11. Uncropped gels corresponding to Supplementary Figure 11A and B. Rectangles indicate the regions shown in the main figures.

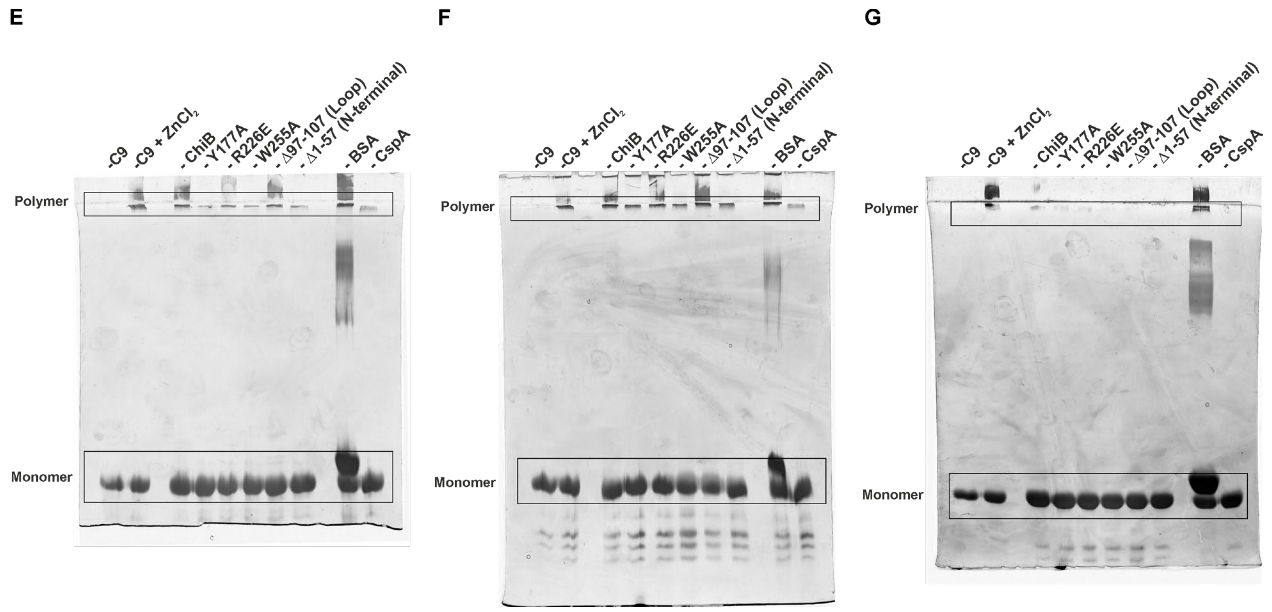

Supplementary Figure S12. Uncropped gels corresponding to Supplementary Figure 12E–G. Rectangles indicate the regions shown in the main figures.

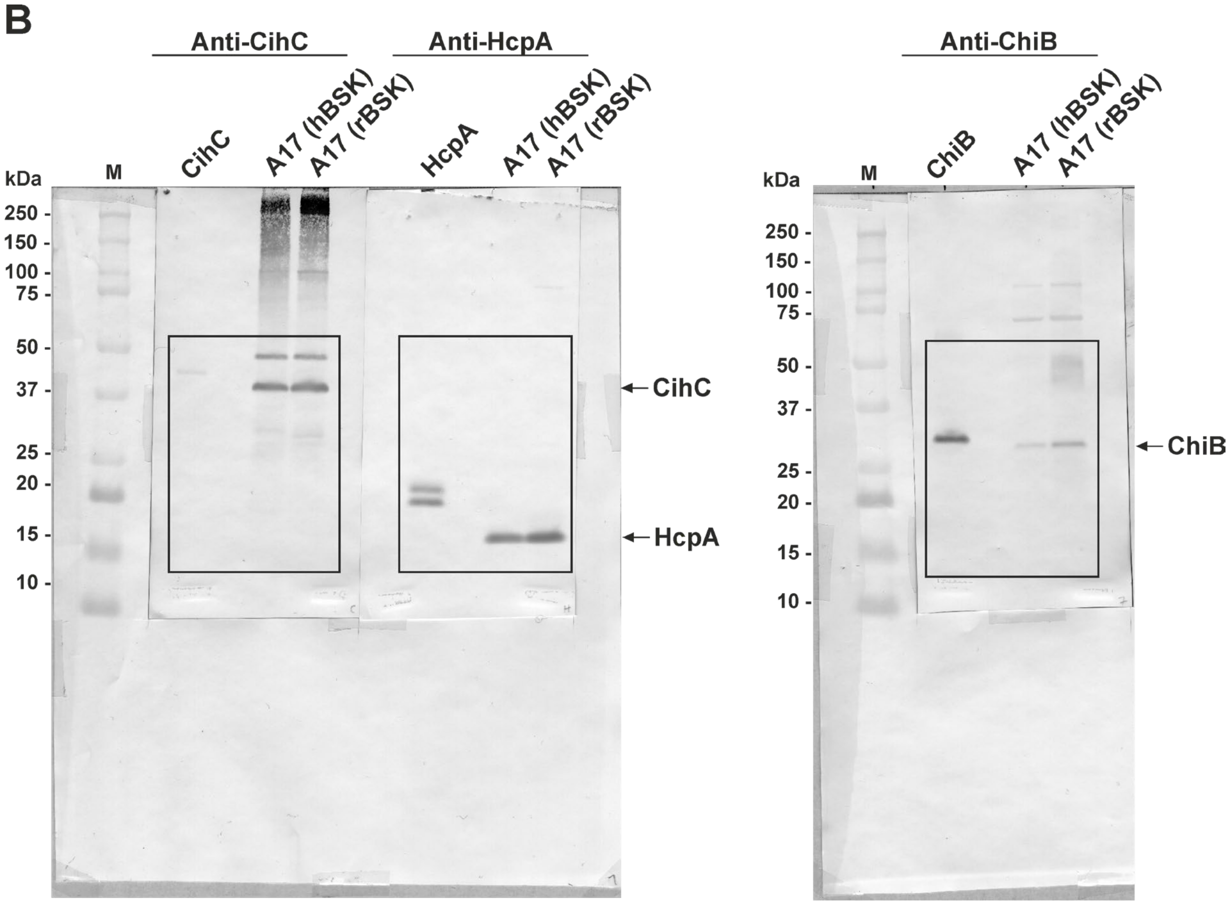

Supplementary Figure S15. Original scans of Western blot membranes corresponding to Supplementary Figure 15B. Rectangles indicate the regions shown in the main figures.

.

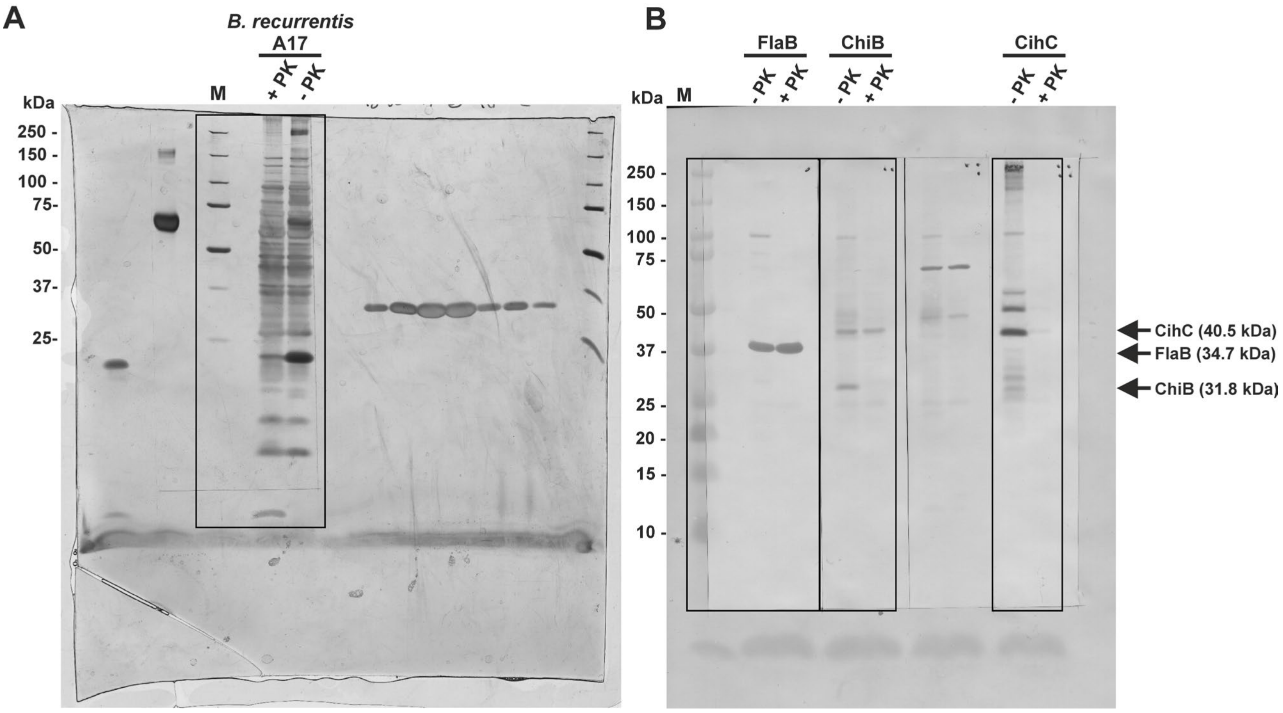

Supplementary Figure S17. Original scans of Western blot membranes corresponding to Supplementary Figure 17A and B. Rectangles indicate the regions shown in the main figures
