# Supplementary material for: Complement inhibition by a unique cluster of immunomodulatory outer surface proteins of Borrelia recurrentis
Source: Nat Commun. 2026 Apr 29;17:3900. doi: 10.1038/s41467-026-72359-y (PMC13128870; doi:10.1038/s41467-026-72359-y)
Supplement: Supplementary file 2 — Reporting summary [file 41467_2026_72359_MOESM2_ESM.pdf]

nature portfolio

Corresponding author(s): Karin Fritz-Wolf

Last updated by author(s): Oct 20, 2025

Reporting Summary

Nature Portfolio wishes to improve the reproducibility of the work that we publish. This form provides structure for consistency and transparency in reporting. For further information on Nature Portfolio policies, see our Editorial Policies and the Editorial Policy Checklist.

Statistics

For all statistical analyses, confirm that the following items are present in the figure legend, table legend, main text, or Methods section.

n/a	Confirmed	
☐	☒	The exact sample size (*n*) for each experimental group/condition, given as a discrete number and unit of measurement
☐	☒	A statement on whether measurements were taken from distinct samples or whether the same sample was measured repeatedly
☐	☒	The statistical test(s) used AND whether they are one- or two-sided<br>*Only common tests should be described solely by name; describe more complex techniques in the Methods section.*
☒	☐	A description of all covariates tested
☐	☒	A description of any assumptions or corrections, such as tests of normality and adjustment for multiple comparisons
☐	☒	A full description of the statistical parameters including central tendency (e.g. means) or other basic estimates (e.g. regression coefficient) AND variation (e.g. standard deviation) or associated estimates of uncertainty (e.g. confidence intervals)
☐	☒	For null hypothesis testing, the test statistic (e.g. *F*, *t*, *r*) with confidence intervals, effect sizes, degrees of freedom and *P* value noted<br>*Give P values as exact values whenever suitable.*
☒	☐	For Bayesian analysis, information on the choice of priors and Markov chain Monte Carlo settings
☒	☐	For hierarchical and complex designs, identification of the appropriate level for tests and full reporting of outcomes
☒	☐	Estimates of effect sizes (e.g. Cohen's *d*, Pearson's *r*), indicating how they were calculated

Our web collection on statistics for biologists contains articles on many of the points above.

Software and code

Policy information about availability of computer code

Data collection	Gen5 (Version 3.16, Agilent BioTek); GraphPad Prism 10 (Version 10.6.0, GraphPad Software); Image Lab (Version 6.1.0, Bio-Rad); LineregPCR (Roche)<br>X-ray diffraction data: SX10SA (detector: Pilatus or EIGER2 16M) of the Swiss Light Source in Villigen, Switzerland. Data were processed using XDS
Data analysis	XDS:data processing, data scaling; PHENIX (AutoSol, AutoBuild, Refine):phasing and structure refinement; COOT:manual model building; SWISS-MODEL:homology modeling; AlphaFold2:structure prediction; UCSF Chimera:molecular graphics and structural analysis; Prokka 1.14.6, Lalign, BLAST, Clustal Omega; iTOL, CLC Sequence Viewer 8, SnapGene 7 (Sequence analyses)

For manuscripts utilizing custom algorithms or software that are central to the research but not yet described in published literature, software must be made available to editors and reviewers. We strongly encourage code deposition in a community repository (e.g. GitHub). See the Nature Portfolio guidelines for submitting code & software for further information.

Data

Policy information about availability of data

All manuscripts must include a data availability statement. This statement should provide the following information, where applicable:

- Accession codes, unique identifiers, or web links for publicly available datasets
- A description of any restrictions on data availability
- For clinical datasets or third party data, please ensure that the statement adheres to our policy

ChiA and ChiB structures are deposited in the Protein Data Bank under accession codes 28LI (ChiA) and 28LK (ChiB) (https://doi.org/10.2210/pdb28LI/pdb; https://doi.org/10.2210/pdb28LK/pdb).
All other data supporting the findings of this study are available within the paper and its Supplementary Information files. Source Data are provided with this paper.
All software used is commercially available or open source, as cited in the Methods section.

Research involving human participants, their data, or biological material

Policy information about studies with human participants or human data. See also policy information about sex, gender (identity/presentation), and sexual orientation and race, ethnicity and racism.

Reporting on sex and gender	Human serum was collected from deidentified healthy blood donors to form a serum pool. All sera used in this study were anonymized after collection. Thus, no sex or gender information has been linked to individual serum samples
Reporting on race, ethnicity, or other socially relevant groupings	Human serum was collected from healthy blood donors to form a serum pool. All sera used in this study were anonymized after collection. Thus, no information of race, ethnicity, or other socially relavant groupings has been linked to individual serum samples.
Population characteristics	Human serum was collected from volunteers (max. 10) to form a serum pool. Thus, no information of population characteristics has been linked to individual serum samples.
Recruitment	Volunteers were asked to donate serum for the study. All participants provided a written informed consent in accordance with the Declaration of Helsinki.
Ethics oversight	Collection of blood samples and consent documents were approved by the ethics committee at the University Hospital of Frankfurt (control number 160/10 and 222/14), Goethe University of Frankfurt am Main.

Note that full information on the approval of the study protocol must also be provided in the manuscript.

Field-specific reporting

Please select the one below that is the best fit for your research. If you are not sure, read the appropriate sections before making your selection.

[x] Life sciences [ ] Behavioural & social sciences [ ] Ecological, evolutionary & environmental sciences

For a reference copy of the document with all sections, see nature.com/documents/nr-reporting-summary-flat.pdf

Life sciences study design

All studies must disclose on these points even when the disclosure is negative.

Sample size	ELISA data shown in this study represent means from at least three experiments where each experiment was performed in triplicates to attain statistical significance ($p<0.05$).
Data exclusions	No data were excluded in this study
Replication	All biological replicates have been presented in this study
Randomization	Regarding ELISA, each protein analyzed was allocated in an individual experimental group where each experiment contains well-characterized controls as standards on the same plate
Blinding	Different investigators were involved in data collection to garantuee blinding

Reporting for specific materials, systems and methods

We require information from authors about some types of materials, experimental systems and methods used in many studies. Here, indicate whether each material, system or method listed is relevant to your study. If you are not sure if a list item applies to your research, read the appropriate section before selecting a response.

Materials & experimental systems

n/a	Involved in the study	
☐	☒	Antibodies
☒	☐	Eukaryotic cell lines
☒	☐	Palaeontology and archaeology
☒	☐	Animals and other organisms
☒	☐	Clinical data
☒	☐	Dual use research of concern
☒	☐	Plants

Methods

n/a	Involved in the study	
☒	☐	ChIP-seq
☒	☐	Flow cytometry
☒	☐	MRI-based neuroimaging

Antibodies

Antibodies used	Anti-C1q (No. A301, Quidel); Anti-C5 (A306, Quidel); Anti-Factor B (A311, Quidel); Anti-Factor I (A313, Quidel); Anti-SC5b-9 (A239, Quidel), Anti-C5 (A220, CompTech); Anti-C3 (No. 204869, Calbiochem, Merck); Anti-C4 (No. 204894, Calbiochem, Merck); Factor H (No. 341276, Calbiochem, Merck); Anti-Plasminogen (No. TA314352, OriGene Technologies); Anti-CihC (Grosskinsky et al., 2010, doi10.1371/journal.pntd.0000698); Anti-HcpA (Schott et al., 2009, doi: 10.1371/journal.pone.0004858); Anti-ChiB (Eurogentec)
Validation	Information of the validation of commercial anti-complement antibodies are available on the following manufacturer`s webpages: Quidel (https://www.quidelortho.com/be/en/products/monoclonal-antibodies-research-only); CompTech (Information are given on each data sheet, https://www.complementtech.com/catalog/complement-polyclonal-antisera/); OriGene Technologies (https://www.origene.com/products/antibodies); Merck (https://www.merckmillipore.com/DE/de/life-science-research/antibodies-assays/antibodies-overview/Antibody-Development-and-Validation/cFOb.qB.8McAAAFOb64qQvSS,nav). For Anti-CihC and anti-HcpA, information is available in the following publications: Grosskinsky et al., 2010, doi10.1371/journal.pntd.0000698 and Schott et al., 2009, doi: 10.1371/journal.pone.0004858). Regarding the Anti-ChiB, purified His-tagged ChiB was used for the generation of a polyclonal rabbit anti-ChiB antiserum by a commercial provider (Eurogentec). Cross-reactivty was tested by Western blotting using whole cell lysates from Borrelia recurrentis A17 and PAbJ (see article).

Plants

Seed stocks	n/a
Novel plant genotypes	n/a
Authentication	n/a
